# *Acanthamoeba* spp. aggregate and encyst on contact lens material increasing resistance to disinfection

**DOI:** 10.3389/fmicb.2022.1089092

**Published:** 2022-12-19

**Authors:** Allison Campolo, Reed Pifer, Rhonda Walters, Megan Thomas, Elise Miller, Valerie Harris, Jamie King, Christopher A. Rice, Paul Shannon, Brian Patterson, Monica Crary

**Affiliations:** ^1^Alcon Research, LLC, Fort Worth, TX, United States; ^2^Department of Comparative Pathobiology, College of Veterinary Medicine, Purdue University, West Lafayette, IN, United States; ^3^Purdue Institute for Drug Discovery (PIDD), Purdue University, West Lafayette, IN, United States; ^4^Purdue Institute of Inflammation, Immunology and Infectious Disease (PI4D), Purdue University, West Lafayette, IN, United States

**Keywords:** *Acanthamoeba*, aggregate, spheroid, cyst, contact lens, contact lens solution

## Abstract

**Introduction:**

*Acanthamoeba* keratitis is often caused when *Acanthamoeba* contaminate contact lenses and infect the cornea. *Acanthamoeba* is pervasive in the environment as a motile, foraging trophozoite or biocide-resistant and persistent cyst. As contact lens contamination is a potential first step in infection, we studied *Acanthamoeba’s* behavior and interactions on different contact lens materials. We hypothesized that contact lenses may induce aggregation, which is a precursor to encystment, and that aggregated encystment would be more difficult to disinfect than motile trophozoites.

**Methods:**

Six clinically and/or scientifically relevant strains of *Acanthamoeba* (ATCC 30010, ATCC 30461, ATCC 50370, ATCC 50702, ATCC 50703, and ATCC PRA-115) were investigated on seven different common silicone hydrogel contact lenses, and a no-lens control, for aggregation and encystment for 72 h. Cell count and size were used to determine aggregation, and fluorescent staining was used to understand encystment. RNA seq was performed to describe the genome of *Acanthamoeba* which was individually motile or aggregated on different lens materials. Disinfection efficacy using three common multi-purpose solutions was calculated to describe the potential disinfection resistance of trophozoites, individual cysts, or spheroids.

**Results:**

*Acanthamoeba* trophozoites of all strains examined demonstrated significantly more aggregation on specific contact lens materials than others, or the no-lens control. Fluorescent staining demonstrated encystment in as little as 4 hours on contact lens materials, which is substantially faster than previously reported in natural or laboratory settings. Gene expression profiles corroborated encystment, with significantly differentially expressed pathways involving actin arrangement and membrane complexes. High disinfection resistance of cysts and spheroids with multi-purpose solutions was observed.

**Discussion:**

Aggregation/encystment is a protective mechanism which may enable *Acanthamoeba* to be more disinfection resistant than individual trophozoites. This study demonstrates that some contact lens materials promote *Acanthamoeba* aggregation and encystment, and *Acanthamoeba* spheroids obstruct multi-purpose solutions from disinfecting *Acanthamoeba*.

## Introduction

*Acanthamoeba* keratitis (AK) is a serious ocular infection that is extremely difficult to treat and can lead to blindness (Siddiqui and Khan, 2012; Szentmary et al., 2019). Currently, AKANTIOR® (polyhexanide; PHMB) at 0.08% concentration is the only drug approved by the FDA (as an orphan drug designation) for *Acanthamoeba* keratitis (Pharma Boardroom, 2022). *Acanthamoeba* is a free-living protist that is pervasive in the environment, and often found in soil and water. Critically, this amoeba is not only commonly found in tap water specifically, but transmission *via* tap water and contact lens association has been linked to the leading causes of AK in Western countries (Carnt et al., 2018, 2020). There are significant education campaigns to inform contact lens wearers of the importance of avoiding water on their contact lenses at all times (Arshad et al., 2019, 2021; British Contact Lens Association, 2021). This amoeba is frequently introduced into the eye *via* contact lenses (Siddiqui and Khan, 2012; Randag et al., 2019), either as the result of inadequate contact lens hygiene habits or due to an ineffective multi-purpose solution (MPS; Verani et al., 2009; Tu and Joslin, 2010; Brown et al., 2018; Carnt et al., 2018). Data indicates that AK cases are increasing, including recent outbreaks in Western countries (Antonelli et al., 2018; Carnt et al., 2018; Randag et al., 2019) which were generally found to be the result of product-specific low *Acanthamoeba* MPS disinfection efficacy (Verani et al., 2009; Yoder et al., 2012). These outbreaks and the incidence rate of AK associated with contact lens users highlight the critical importance of adequate MPS disinfection efficacy against *Acanthamoeba*. While poor MPS disinfection efficacy is often blamed for *Acanthamoeba* infections, it is possible that contact lens materials themselves play an important role in the potential of *Acanthamoeba* to infect the eye. While *Acanthamoeba* trophozoites and cysts will bind to a wide variety of polymeric surfaces (Kilvington and Larkin, 1990; Beattie et al., 2011), the differences in silicone hydrogel contact lens materials have not been considered as playing a role in *Acanthamoeba* pathogenesis. Thus, contact lenses may have inappropriately avoided blame by being recognized as a mere vector in the path to *Acanthamoeba* keratitis infection, as opposed to having an impact on the potential for a corneal infection.

*Acanthamoeba* exists either in the motile, infective trophozoite form or as the more resistant, persistent cyst form, which can remain viable for years (Mazur et al., 1995; Siddiqui and Khan, 2012). Cysts are notoriously difficult to eradicate versus the trophozoite form, and have been shown to be impervious to most disinfection methods that do not involve hydrogen peroxide or povidone iodine (Johnston et al., 2009; Coulon et al., 2010; Ahearn et al., 2012; Walters et al., 2022). While not generally considered a social amoebae like *Dictyostelium*, which can become a multicellular structure during their lifecycle, *Acanthamoeba* has been shown in the literature as forming clumps of cysts. This social behavior has not been studied significantly though *Acanthamoeba* aggregation has been observed during viral infection of the amoeba (Oliveira et al., 2019) as well as a precursor to encystment (Coulon et al., 2010). Both mechanisms suggest a protective action similar to that seen in *Dictyostelium* where the multicellular structure differentiates with some amoeba becoming cysts and others sacrificing themselves to form the protective fruiting body (Kilvington et al., 2009; Schaap, 2011; Kilvington and Lam, 2013; Oliveira et al., 2019). *Acanthamoeba* aggregation as a precursor to encystment has an evolutionary advantage that would allow protection of interior trophozoites from the environmental trigger promoting encystment. However, *Acanthamoeba* aggregation has not been studied outside of viral infection and many *Acanthamoeba* investigations identify that *Acanthamoeba* cysts are observed as clumps or spheroids but provide no hypothesis on the biological mechanisms occurring. Spheroids can be made of either trophozoites or cysts (Griffiths, 1969; Coulon et al., 2010; Ahearn et al., 2012) and it is currently unknown how aggregation affects cyst adherence to contact lenses. The underlying genes associated with aggregation remain largely unknown and the cellular pathways involved in encystment are still being described (Rolland et al., 2020). Encystment occurs when *Acanthamoeba* identifies the environment as unfavorable but any number of triggers from temperature, osmolarity, or nutrient availability, have been associated with encystment (Lloyd, 2014; Mahboob et al., 2016).

Encystment is a patient safety risk as *Acanthamoeba* cysts are difficult to kill both when they are on contact lenses and when they are in the cornea (Rayamajhee et al., 2021). One *Acanthamoeba* keratitis outbreak was specifically associated with a multi-purpose solution that induced encystment of *Acanthamoeba* trophozoites and failed to effectively kill *Acanthamoeba* cysts (Verani et al., 2009). This allowed *Acanthamoeba* to be transferred to the eye *via* contact lenses where *Acanthamoeba* then excysted and became pathogenic. Previous research on *Acanthamoeba*’s interaction with contact lenses has focused on its rate of adherence and the number of *Acanthamoeba* that can form strong bonds to the surface of contact lenses (Kilvington and Larkin, 1990; Ibrahim et al., 2009; Lee et al., 2018). Unfortunately, many of the published results in this field are contradictory and little consensus can be found in the literature on which contact lenses demonstrate the most abundant *Acanthamoeba* adherence (John et al., 1989; Kilvington, 1993; Seal et al., 1995; Lee et al., 2016). Meanwhile, the contact lens industry continues to expand with new materials and surface chemistries (Musgrave and Fang, 2019). Here, we developed new methods to observe and quantify the behavior of six different potentially keratitis-causing *Acanthamoeba* strains on contact lens materials to determine if the *Acanthamoeba* response to different materials could possibly play a role in *Acanthamoeba* transmission to the eye. We observed that *Acanthamoeba* aggregates and encysts in response to some lens materials, independent of MPS exposure. To understand the mechanisms by which contact lenses contribute to aggregation, we evaluated the altered gene expression of *Acanthamoeba* when in contact with different lens materials and identified genes which may be critical to this material-specific aggregation process. Finally, we evaluated resistance to multi-purpose solution disinfection when *Acanthamoeba* are aggregated. Thus, we show here not only an extremely robust investigation into the behavior and motility of this pervasive pathogenic amoeba, but we also show for the first time that contact lens materials may play a critical role in increasing the risk of *Acanthamoeba* keratitis and affecting patient safety through disinfection resistance.

## Materials and methods

### *Acanthamoeba* culturing

*Acanthamoeba* strains were obtained from ATCC (American Type Culture Collection, Manassas, VA). Strains used and their information can be found in Table 1.

**Table 1:** Description of the strains of *Acanthamoeba* used (de Lacerda and Lira, 2021) contact lens material tested, and multi-purpose solutions used.

Acanthamoeba	Genotype	Strain	Keratitis-causing genotype	Original source
ATCC 50702	T3	TIO:H37	Yes (Sawyer, 1971; Chelkha et al., 2020)	Keratitis
ATCC 30461	T4	Eye	Yes (Acanthamoeba polyphaga (Pushkarew), 2019; Pushkarew, 1913)	Keratitis
ATCC 50370	T4	Ma	Yes (Douglas, 1930; Gatti et al., 1998)	Keratitis
ATCC 30010	T4	Neff	Yes (Neff, 1957; Chelkha et al., 2020)	Environment
ATCC 50703	T5	45	Yes (Molet and Ermolieff, 1976; Cruz and Rivera, 2014)	Human Nose
ATCC PRA-115	T11	4RE	Yes (Sawyer et al., 1977; Gast, 2001)	Lens case
**Contact lens material**	**Brand name**	**Manufacturer**		**Group/Water content**
omafilcon B	ProClear	CooperVision, San Ramon, CA, USACooperVision, San Ramon, CA, USACooperVision, San Ramon, CA, USA		2
comfilcon A	Biofinity			5C/48%
fanfilcon A	Avaira Vitality			5B /55%
samfilcon A	Ultra	Bausch + Lomb® Rochester, NY, USA		5C/46%
etafilcon A	Acuvue 2	Johnson & Johnson Vision Care, Jacksonville, FL, USAJohnson & Johnson Vision Care, Jacksonville, FL, USA		4
senofilcon A	Acuvue Oasys			5C/38%
lehfilcon A	TOTAL30	Alcon® Fort Worth, TX, USA		5B/55%
**Multi-purpose solution biocide composition**		**Brand name**	**Manufacturer**	**Disinfection time**
Polyaminopropyl biguanide (0.00013%), (**PAPB**)		Lite	CooperVision, San Ramon, CA, USA	6 h
Polyaminopropyl Biguanide Hydrochloride (0.00013%), polyquaternium (0.0001%) (**PAPB/PQ**)		Biotrue®	Bausch + Lomb® Rochester, NY, USA	4 h
Polyaminopropyl biguanide (0.00013%), polyquaternium (0.0001%), alexidine dihydrochloride (0.00016%) (**PAPB/PQ/AD**)		Biotrue® Hydration Plus	Bausch + Lomb® Rochester, NY, USA	4 h

As previously described (Walters et al., 2022), trophozoites were axenically cultured in AC6 media (axenic culture medium, containing 20 g biosate peptone, 5 g glucose, 0.3 g KH_2_PO_4_, 10 μg vitamin B12, and 15 mg L-methionine per liter of distilled deionized water). Media was adjusted to a pH of 6.6–6.95 with 1 M NaOH and autoclaved at 121°C for 20 min before storing at room temperature for use within 3 months. ¼ Ringer’s solution was used to harvest organisms. To create a homogenous population of *Acanthamoeba* trophozoites, *Acanthamoeba* were scaled up in fresh AC6 media 24 h to testing. Cells were then collected and centrifuged at 500 g for 5 minutes, followed by a wash and resuspension using ¼ Ringer’s solution. Count seeding was confirmed *via* manual counting using a hemocytometer.

### Contact lenses and mutli-purpose solutions used

Information about contact lenses and multi-purpose solutions used and their details can be found in Table 1. Multi-purpose solutions tested were chosen by their representation of popular multi-purpose solutions and are identified by biocide throughout the manuscript: PAPB/PQ [polyaminopropyl biguanide (0.00013%), polyquaternium (0.0001%)], PAPB/PQ/AD [polyaminopropyl biguanide (0.00013%), polyquaternium (0.0001%), alexidine dihydrochloride (0.00016%)], and PAPB [polyaminopropyl biguanide (0.00013%)]. Lenses were always paired by power for each replicate of an experiment (that is, for each replicate, every lens would be of the same power to reduce variability). Lenses were acquired based on market availability. For aggregation quantification, −12 power lenses were used. For RNA collection, −12 and −6 power lenses were used. For confocal experiments, −3 power lenses were used. All lenses used were recorded visually during the experimental procedure to ensure similar behavioral patterns – power was not observed to impact aggregation.

### *Acanthamoeba* observation and quantification of count and particle size on contact lens materials

**Figure 1:**
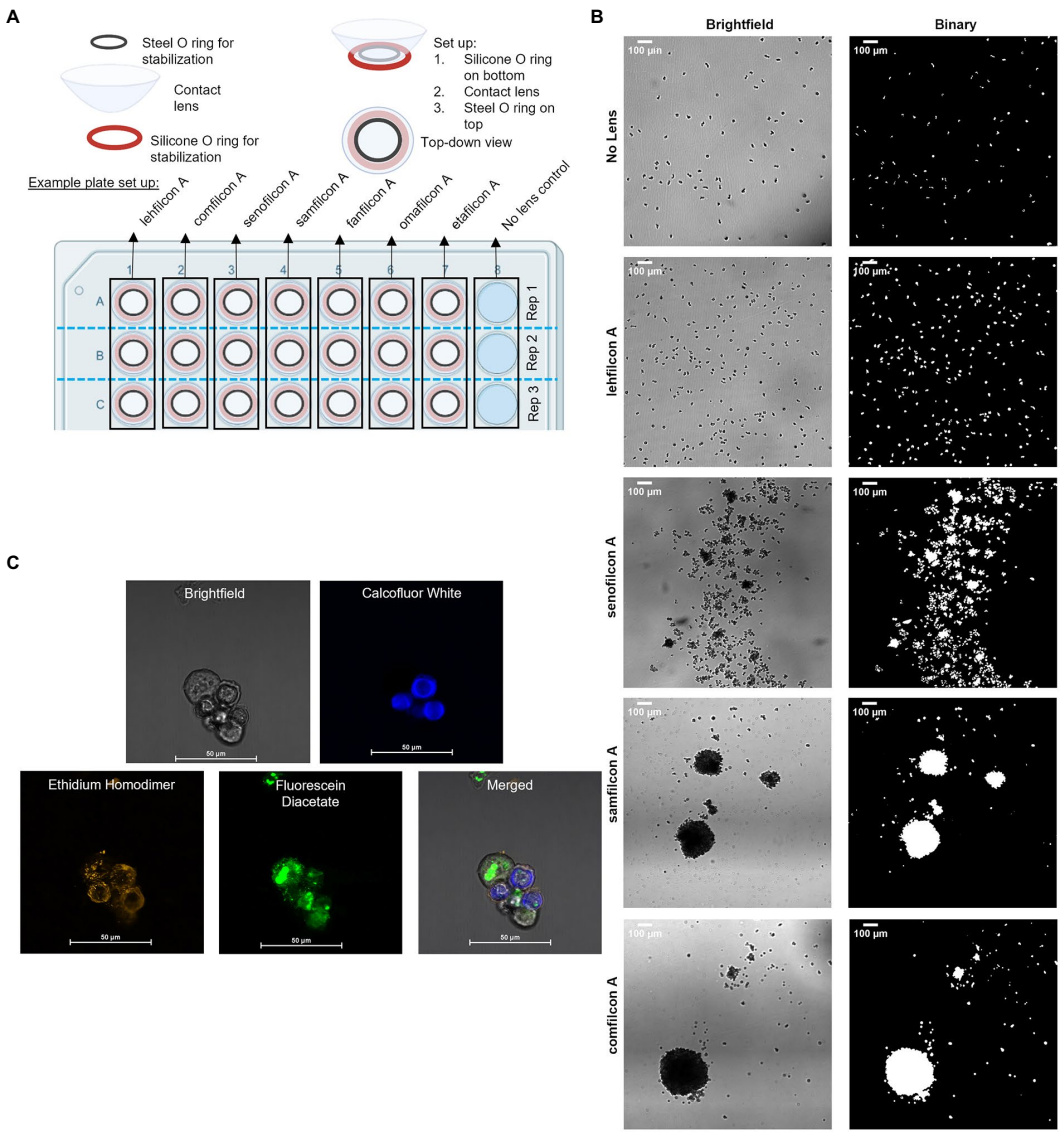
Methodological representations. **(A)** Plate set up for time lapse count and strain analysis of *Acanthamoeba* aggregation on contact lens materials. **(B)** Representative large images taken at 4× magnification in brightfield, and then as they appear in binary which is used for count and size analysis (scale bar = 100 μm). **(C)** Representative large images of fluorescent confocal microscopy images, taken in brightfield, with three different filters, and merged (please refer to Figure 7 for details; scale bar = 50 μm) depicting encystment at 6 h using a Biofloat spheroid plate.

Contact lenses were trimmed to 12 mm utilizing a biopsy punch. In a 48-well plate, a silicone O-ring was placed at the bottom of each well (Figure 1A). The contact lenses were placed on top of the silicone O-ring, then an additional O-ring was placed on top of the contact lens. This allowed the contact lens to maintain its normal curvature but prevented lens floating during extended timelapse observation. 500 μl of ¼ Ringer’s was added to the top of the contact lenses. ~3,000 trophozoites were added to each well containing a contact lens. Amoeba occasionally demonstrated a ring pattern due to a slight wrinkle in the bottom of the lens caused by the round lens being sat on a flat well. A no-lens control (containing both O-rings) was also executed in the same polystyrene plate. The 48-well plate was transferred to a Nikon microscope with motorized stage. *Acanthamoeba* were allowed to settle for 10 min and then each well was imaged using a 2×2 stitched large image (NIS Elements AR 3.2) at 4× magnification for a continuous period of 12 h, with each well being imaged every 3 min. Later timepoints at 24, 48, and 72 h were also conducted for 30 min of continuous imaging with each wellbeing imaged every 3 min. All videos were concatenated such that each contact lens had a single video file containing 274 images representing the entire 72-h period of observation. Seven contact lenses plus a no lens control were executed for each replicate. Six replicates were conducted for each strain of *Acanthamoeba* and six strains of *Acanthamoeba* were utilized.

Timelapse videos were recorded in grayscale using bright-field microscopy. Using ImageJ (version 1.53q), videos were converted into a high-contrast, binary format for analysis (Figure 1B). Briefly, image thresholding as determined by ImageJ was used to convert greyscale images into binary. Non-amoebic artifacts were removed utilizing fill and clear functions within ImageJ as needed. Particle analysis was conducted on the binary images, which included count and size of all particles (amoeba) within a frame. Individual images were created by duplicating frames within the timelapse video into new image files as needed. The size and count of each contact lens/replicate/strain were evaluated independently. For each timelapse video, 30-min (10 frame) sections were averaged for size and count across the 72 h. Size counts were normalized such that 100% represented a single individual trophozoite size, and anything over 100% indicates a spheroid of more than one trophozoite combined. This was conducted independently for each video as the image field had slight differences in plane, resulting in variable trophozoite size depending on if the microscope was focused on the top, middle or bottom of the cell. The size and count across the six replicates for a specific contact lens/no lens control were averaged for each strain of *Acanthamoeba*. Count and normalized particle size were graphed as a function of time for each strain.

To allow the amoeba to settle onto the lens and compensate for size differences observed between strains, the size of each strain-lens condition was normalized to its own 0.5–1.0 h reference time (Campolo et al., 2021). Additionally, due to the count difference of the no lens control, which was not confined to a smaller field of view by the bowl of the contact lens, the individual particle count of the no lens control was not included in the statistical analysis. Amoeba size and count within each timepoint within each replicate of each strain and lens combination were averaged, and standard deviation was calculated to identify outliers. Replicates (*n* = 6) of identical conditions were then averaged by timepoint, and standard error of the mean calculated. Normality was assessed using a Shapiro–Wilk test, and size and count (between conditions (lens material) at any timepoint, and within each condition over time) were analyzed *via* two-way repeat measure ANOVA with a post-hoc Tukey’s multiple comparisons test (GraphPad Prism 9.2.0). An alpha of 0.05 was used to assess significance in all comparisons.

### Standard curve spheroid generation

*Acanthamoeba* ATCC 30461 trophozoites were seeded into Biofloat plates (faCellitate, Mannheim, Germany) at a density of 8, 16, 32, 125, 250, 500, 1,000, or 2,000 cells/well in replicates of 8 per plate. The experiment was conducted across 4 independent 96-well plates per time period. Timelapse images were taken of each spheroid every 5 min for 3 h, then every 15 min for the subsequent 6 h, and then every 30 min from hours 9 through 24. Each well made one spheroid and the timelapse videos were converted to binary images in the same fashion as the contact lens videos. Each spheroid video was analyzed to determine the number of trophozoites per spheroid, as well as the area of each spheroid as a function of time. A standard curve was generated as a function of trophozoite count vs. spheroid size over time using spheroid area (Supplementary Figure S1). To validate standard curve cell count estimation method, count estimates using the standard curve were compared against traditional hemocytometer method (Buck and Rosenthal, 1996) and validated across a range of 100–1,500 cells per spheroid.

### Confocal imaging of spheroids

Spheroids were generated on Biofloat (faCellitate, Mannheim, Germany) plates or contact lenses as described above (Figure 1C). Spheroid age was between 2 and 72 h depending on the images. Prior to aggregation experiments, control trophozoites and cysts [pre-generated separately *via* starvation (Walters et al., 2022)] were stained to verify stain response to cells. No control cells were added to aggregation experiments. To investigate spheroid formation, trophozoites on normal tissue culture plates were incubated for the same time period as spheroids as a control. Following spheroid generation, spheroids were stained using calcofluor white (Millipore Sigma, Darmstadt, Germany, Catalog #F1303), ethidium homodimer (ThermoFisher Scientific, Massachusetts, USA, Catalog #E1169), and fluorescein diacetate (ThermoFisher, Catalog #F1303). Spheroids were stained with calcofluor white (blue color) which binds to the cellulose of cell walls and is only present in cysts (Magistrado-Coxen et al., 2019). Ethidium homodimer (orange staining) binds to nucleic acids and indicates a compromised cell wall (cell death) or the formation of an extracellular matrix. Fluorescein diacetate (green color) is a dye that can penetrate cell walls and indicates ongoing enzymatic activity as only living cells will convert the nonfluorescent dye into the green fluorescent compound fluorescein.

### *Acanthamoeba* DNA sequencing

Crude DNA extracts were prepared from ATCC 30461 with a cetyl trimethylammonium bromide-based procedure using Carlson Lysis Buffer (CLB; Bioworld, Dublin, OH, USA, # 10450002; Vaillancourt and Buell, 2019). Briefly, *Acanthamoeba* trophozoites were passaged and collected to create a pellet of 2 × 10^7^ cells. Pellets were resuspended in CLB containing 0.25% 2-mercaptoethanol and 0.7 mg/ml RNase A and incubated at 54°C–56°C for 60 min at 1200 RPM in an Eppendorf Thermomixer R. Proteinase K was added to 7 U/ml and incubated with shaking for an additional 20 min. Two sequential chloroform:isoamyl alcohol extractions were performed, followed by an isopropanol precipitation. Crude extracts were dissolved at 54–56°C in Qiagen G2 buffer containing 0.2 mg/ml RNase A and 15 U/ml Proteinase K and further purified by through a 20/G genomic tip according to the manufacturer’s instructions. DNA purity was assessed by agarose gel electrophoresis and quantified using a Take3 Micro-Volume Plate with a Synergy H4 plate reader and Gen5 Software (Biotek, Winooski, VT, USA). Illumina sequencing, Oxford Nanopore sequencing, and analysis were performed by Seqcenter (Pittsburgh, PA, USA). Quality control and adapter trimming was performed with bcl-convert (2021) and rrwick/Porechop, Github.Com (2017) for Illumina and ONT sequencing, respectively. Long read assembly with ONT reads was performed with Flye (Lin et al., 2016). The long read assembly was polished with Pilon (Walker et al., 2014). To reduce erroneous assembly artifacts caused by low quality nanopore reads, long read contigs with an average short read coverage of 15x or less were removed from the assembly. Assembly statistics were recorded with QUAST (Gurevich et al., 2013). Assembly annotation was performed with Funannotate (Jon and Jason, 2019).

### *Acanthamoeba* RNA harvesting and sequencing

In a 24-well plate, contact lenses were place concave side up in each well. 100 μl of ¼ Ringer’s solution was placed below the lens to keep it supported and moist, and the lid was secured to the plate to prevent drying. 75 μl of ¼ Ringer’s suspending 5 × 10^4^ *Acanthamoeba* was placed onto the upward-facing concave side. Wells were imaged continuously at one image every 24 s while amoeba were on the lens to ensure that lenses were centered, amoeba were on top of the lens, and amoeba exhibited similar behavior as seen in *Acanthamoeba* quantification experiments. At the end of the specified time period, amoeba were harvested without disturbing the lenses by pipetting amoeba off and pipette-washing the lens with 50 μl ¼ Ringer’s. All wells within a technical replicate were combined in a singular sample collection tube for a total minimum of 5×10^5^ cells per sample. Lenses and wells were examined *via* microscope after harvesting to ensure all amoeba were collected and none remained in the well. *Acanthamoeba castellanii* (ATCC 30461) samples were collected from the lenses directly into TRIzol (ThermoFisher, Waltham, MA, USA, #15596026) and RNA was isolated immediately using the PureLink RNA Micro Scale Kit (ThermoFisher, #12183016). RNA integrity was assessed by agarose gel electrophoresis and quantified using a Take3 Micro-Volume Plate with a Synergy H4 plate reader and Gen5 Software. For each condition and time point, six independent replicates were prepared on separate days. RNA sequencing and analysis was performed by Seqcenter. Samples were then DNase treated with Invitrogen DNase (RNase free). Library preparation was performed using Illumina’s Stranded Total RNA Prep Ligation with Ribo-Zero Plus kit and 10 bp IDT for Illumina indices. Sequencing was done on a NextSeq2000 giving 2x50bp reads. Quality control and adapter trimming was performed with bcl-convert v3.9.3 (2021). Read mapping was performed *via* STAR (Dobin et al., 2012) using the previously sequenced genome of ATCC 30461 as reference. Feature quantification was performed using RSEM (Li and Dewey, 2011). Read counts loaded into R and were normalized using edgeR’s (Robinson et al., 2009) Trimmed Mean of M values (TMM) algorithm. Subsequent values were then converted to counts per million (cpm). Differential expression analysis was performed using edgeR’s Quasi-Linear F-Test (qlfTest) functionality against treatment groups. Differentially expressed genes were considered those with |log2FC| > 1 and *p* < 0.05.

Affinity Propagation clustering (17218491) of RNA sequencing results was performed in Python version 3.8.8 using scikit-learn version 0.24.1. Parameters used were 0.5 damping, a maximum of 200 iterations, 15 unchanged iterations until convergence. The dimensional inputs for Euclidean distance-based affinity propagation were composed of the following gene expression comparisons: lehfilcon A vs. polystyrene control at 4, 12, or 24 h, samfilcon A vs. polystyrene control at 4, 12, or 24 h, and comfilcon A vs. polystyrene control at 4, 12, or 24 h. The average log2 fold changes in gene expression from six RNA sequencing replicates were used as value inputs for each dimension. Only significantly differentially expressed genes were included in analysis. The resulting gene clusters were further reduced into phenotypic clusters by correlational distance-based affinity propagation on the median expression change of all genes included in each primary cluster. Heatmaps of the resulting gene subsets were constructed in GraphPad Prism 9.2.0.

Locus tags from the genomic database created by DNA sequencing (of the ATCC 30461 strain, internal identifiers from these datasets are FUN_*) and the associated information with each tag was used to identify homologs and inferred gene function based on the known function of the ATCC 30010 strain. The amino acid sequence of each ATCC 30461 gene was used to search NCBI’s BLAST (National Center for Biotechnology Information, Basic Local Alignment Search Tool) to determine percent homology with known genes of all species. As homology with ATCC 30010 was most prevalent, this and the associated Neff strain locus tag were used to estimate gene function. Neff strain locus tags (ACA1_*) were searched in the AmoebaDB informatics resource repository to define the GO (Gene Ontology) terms for each gene. Neff strain locus tags were also used to identify the associated protein ID in either the UniProt or KEGG (Kyoto Encyclopedia of Genes and Genomes) databases, and significant common pathways were identified using STRING (Search Tool for the Retrieval of Interacting Genes/Proteins; false discovery rate < 0.05 using the Benjamini-Hochberg procedure).

### Disinfection efficacy

The disinfection efficacy of individual trophozoites, spheroids, and cysts were evaluated in a disinfection study. The disinfection study was conducted concurrently across conditions with three independent inoculums of *Acanthamoeba* ATCC 30461.

Spheroids: The wells of a 96-well Biofloat plate (faCellitate, Mannheim, Germany) were seeded with a serial dilution of *Acanthamoeba* trophozoites such that wells contained either 100, 375, or 1,000 cells per well.

Cysts: Cysts were generated by starvation on non-nutrient agar plates. Briefly, trophozoites were harvested into ¼ Ringer’s and plated on non-nutrient agar plates and incubated at 28°C for a minimum of 10 days. After incubation, cysts were rinsed from plates using ¼ Ringers and stored at 4°C until testing.

Trophozoites and cysts: The wells off a 96-well flat bottom tissue culture plate were seeded with a serial dilution of *Acanthamoeba* cells such that wells contained either 100, 375, or 1,000 cells per well.

Spheroids, trophozoites, and cysts: Eight replicates of each concentration were conducted per multi-purpose solution and independent inoculum. Cells were incubated in the wells for either 12 or 24 h prior to being exposed to multi-purpose solutions. Excess ¼ Ringer’s was removed from each well, and 200 μl of the designated multi-purpose solution was added to the well. At disinfection time (4 or 6 h), the MPS was removed and 25 μl of Letheen broth was added to each well to neutralize any remaining biocide. The total contents of each well were transferred to a 48-well plate containing 500 μl of non-nutrient agar. Heat-killed *Escherichia coli* was added to each well and the 48-well plates taped and incubated for 21 days at 28°C.

After 21 days, all plates were scored for growth. Each multi-purpose solution/cell type/incubation length/inoculum was quantified as a % outgrowth for a particular condition. Comparisons between cell type, incubation length and multi-purpose solutions were conducted and analyzed *via* 2-way ANOVA, with *post hoc* Tukey’s test (GraphPad Prism 9.2.0). Significance was set at 0.05.

## Results

### *Acanthamoeba* behavior on lens materials

**Figure 2:**
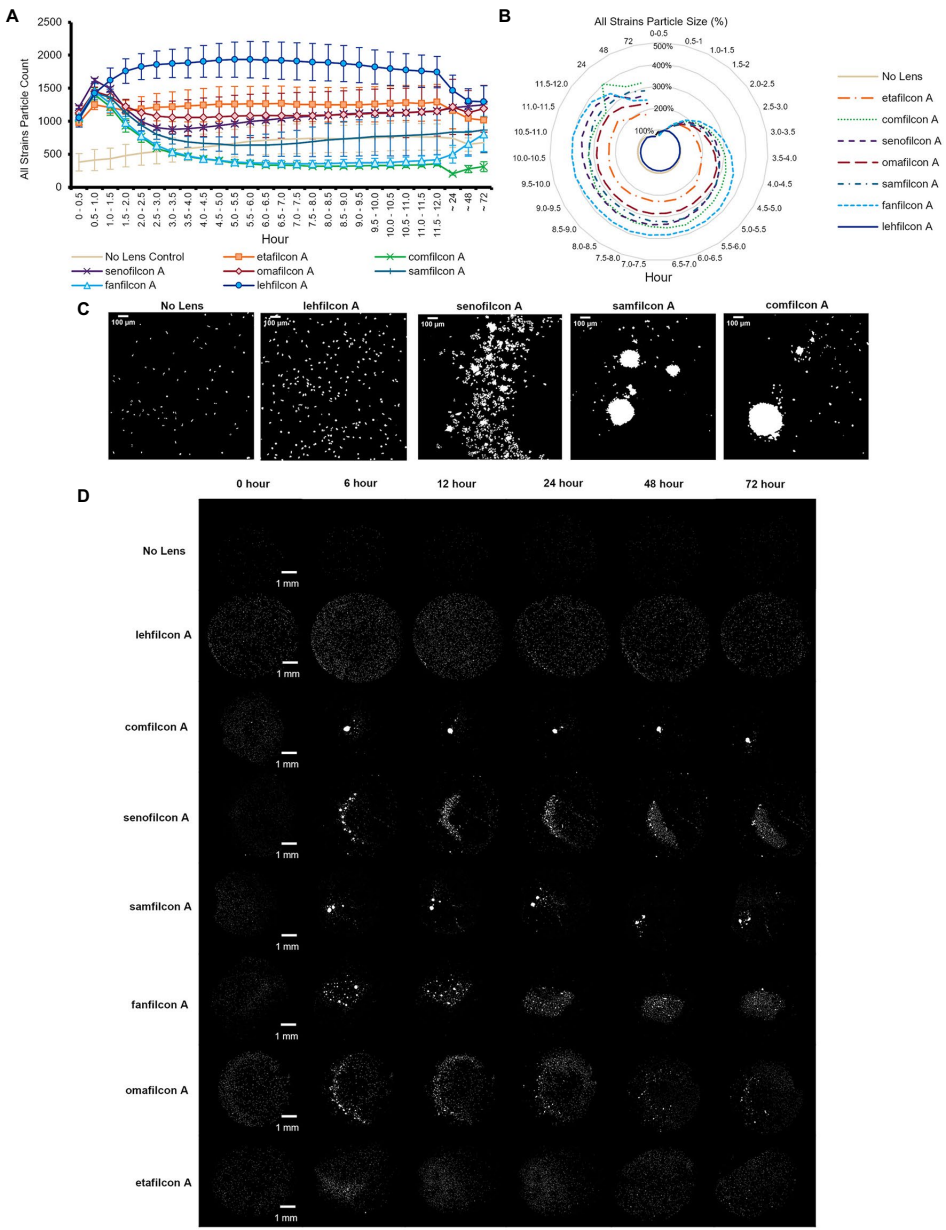
*Acanthamoeba* demonstrated significantly less aggregation on lehfilcon A lenses compared to other materials. Mean ± SE of **(A)** all strains (ATCC 30010, 30461, 50370, 50702, 50703, and PRA-115) count (number of individual particles), **(B)** all strains normalized particle size. **(C)** Enlarged representative binary images of amoeba (ATCC 30461) on contact lenses at 12 h timepoint (scale bar = 100 μm), **(D)** Representative binary images of amoeba (ATCC 30461) on all materials tested (scale bar = 1 mm). Size is normalized to the baselines obtained in the 0.5–1.0 h. Statistical comparisons for subpanels **(A)** and **(B)** noted in Supplementary Figure S2. *n* = 6 per group.

**Figure 3:**
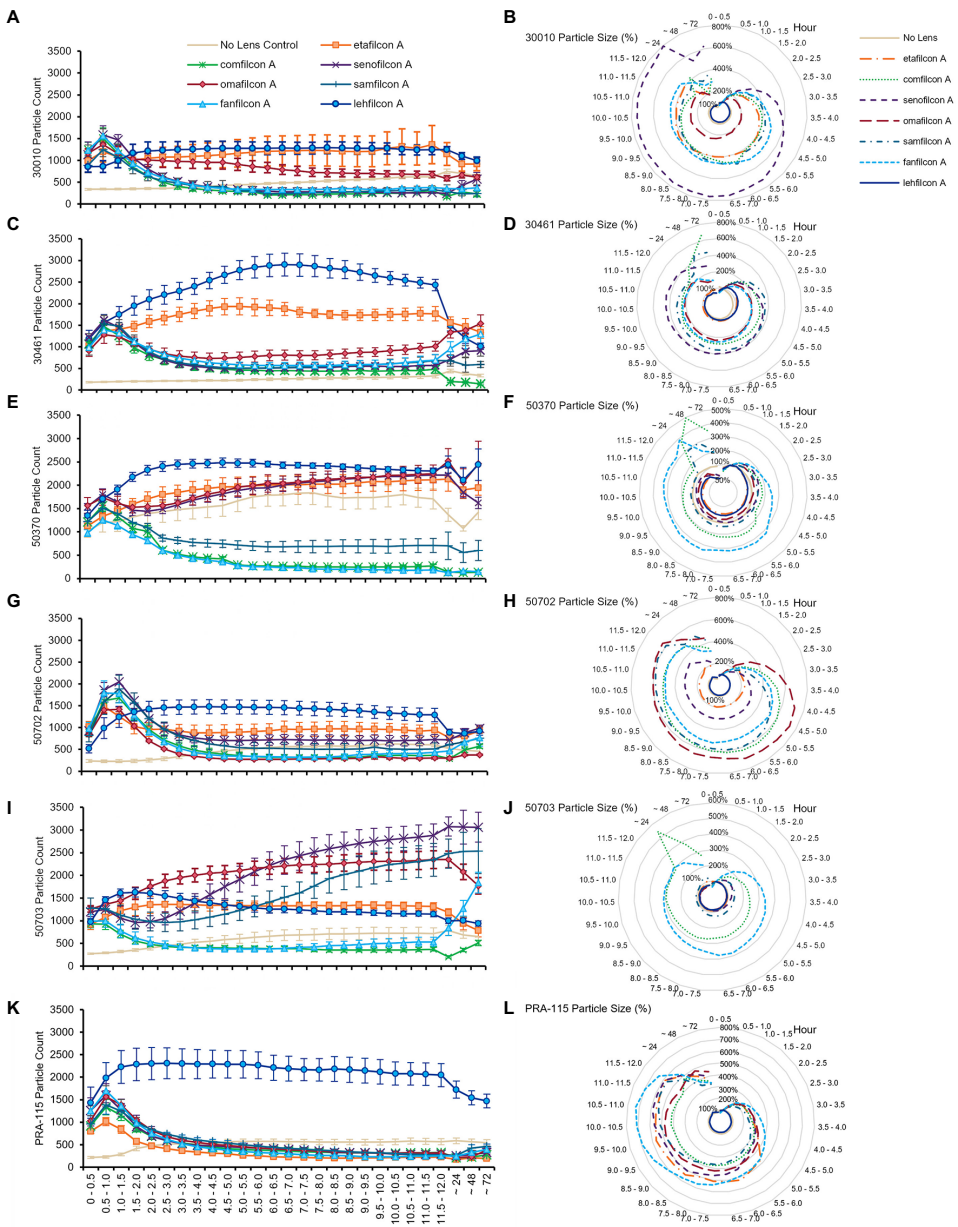
*Acanthamoeba* demonstrated significantly less aggregation on lehfilcon A lenses compared to other lens materials. Mean ± SE of **(A)** ATCC 30010 count, **(B)** ATCC 30010 particle size, **(C)** ATCC 30461 count, **(D)** ATCC 30461 particle size, **(E)** ATCC 50370 count, **(F)** ATCC 50370 particle size, **(G)** ATCC 50702 count, **(H)** ATCC 50702 particle size, **(I)** ATCC 50703 count, **(J)** ATCC 50703 particle size, **(K)** ATCC PRA-115 count, **(L)** ATCC PRA-115 particle size. Size is normalized to the baselines obtained in the 0.5–1.0 h. Statistical comparisons noted in Supplementary Figures S3 and S4. Matching representative images presented in Figures 4 and 5. *n* = 6 per group.

**Figure 4:**
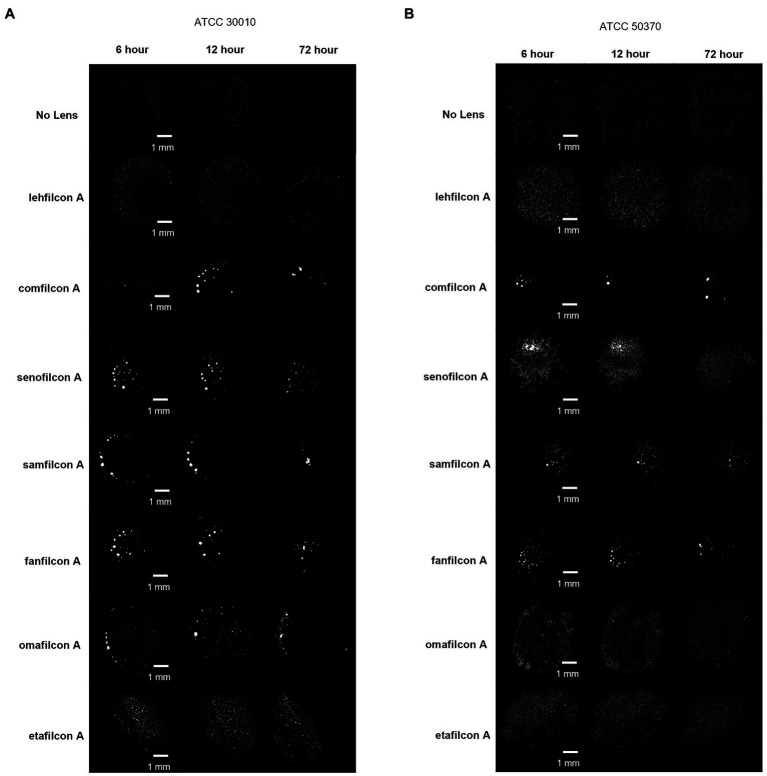
Representative images of *Acanthamoeba* on contact lens materials at 6, 12, and 72 h. **(A)** ATCC 30010, **(B)** ATCC 50370. Mean ± SE representation in Figure 3. *n* = 6 per group.

**Figure 5:**
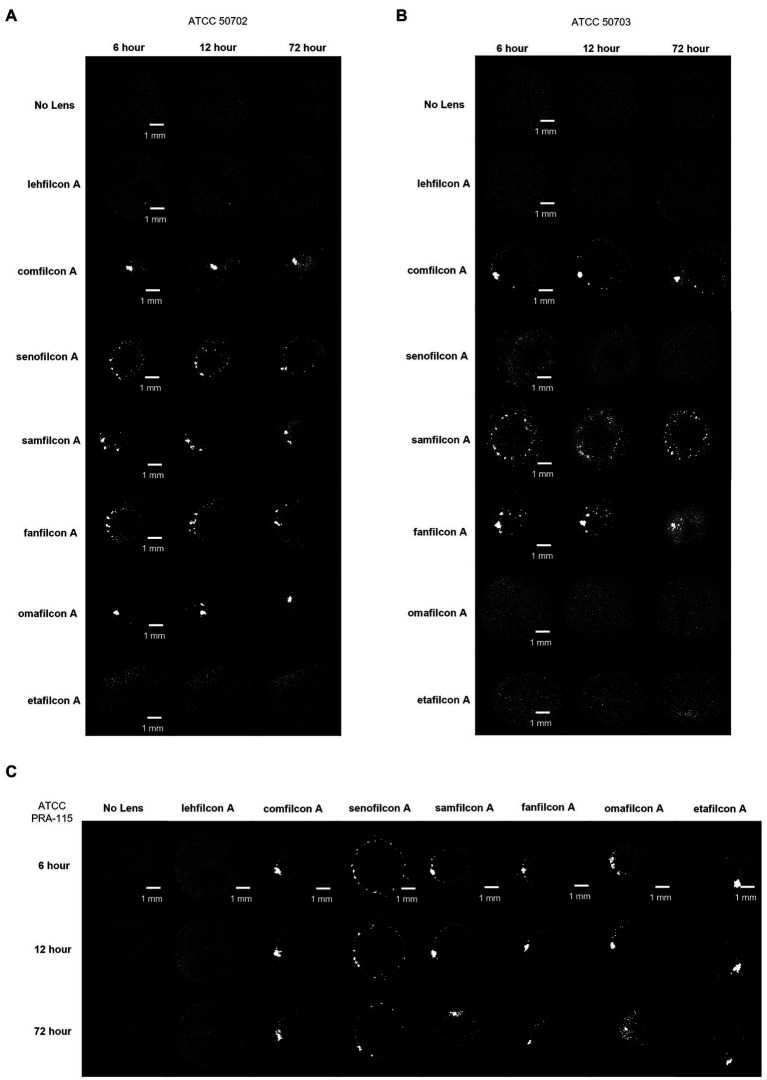
Representative images of *Acanthamoeba* on contact lens materials at 6, 12, and 72 h. **(A)** ATCC 50702, **(B)** ATCC 50703, **(C)** ATCC PRA-115. Mean ± SE representation in Figure 3. *n* = 6 per group.

To understand the differences in *Acanthamoeba* behavior on popular lens materials, we investigated six different potentially keratitis-causing strains of *Acanthamoeba* for 72 h on seven different lens materials, as well as a polystyrene no lens control in which *Acanthamoeba* appeared to move independently and consistently similar to previously examined surfaces (Campolo et al., 2021; Figures 2–5; Supplementary Figures S2–S4). An experimental timeline was designed to allowed us to observe and quantify aggregation both in the clinically normal periods of when a contact lens might be stored individually in a contact lens case overnight, as well as longer periods to determine if any behavior was transient (de-aggregation of *Acanthamoeba* on their own without conditions otherwise changing). To quantify behavior, we determined both the particle count (the number of individual amoeba or spheroids identifiable in the field of view) and the particle size. As spheroids form, an inverse relationship between particle count and average size is observed (i.e., as counts decrease, size increases).

When data from all strains were combined (Figures 2A,B; Supplementary Figure S2), we found that all lens materials tested demonstrated a significantly lower particle count than lehfilcon A (*p* < 0.05) from timepoints 1.5–2.0 h to 9.0–9.5 h, with comfilcon A, senofilcon A, samfilcon A, fanfilcon A, and omafilcon A being significantly lower through at least 12 h. Similarly, with all strains data combined, all lens materials except etafilcon A demonstrated a significantly higher particle size from at least 3.0–3.5 h through 72 h than lehfilcon A (*p* < 0.05). In this analysis, etafilcon A demonstrated a significantly higher particle size than lehfilcon A from 6.0–6.5 h through 11.5–12 h. All lens materials were also analyzed for their change compared to their baseline (0.5–1.0 h) in both particle count and particle size. When all strains were combined it was noted that lehfilcon A, omafilcon A, and etafilcon A did not demonstrate a significant change compared to their particle count baseline, while comfilcon A, senofilcon A, samfilcon A, and fanfilcon A did, beginning by 1.5–2.0 h to 2.0–2.5 h (*p* < 0.05). Some lens materials (comfilcon A, fanfilcon A) maintained this difference through 72 h, while others (senofilcon A, samfilcon A) demonstrated a relative return to their baseline before the end of the experiment. Overall, when combining the data from all six *Acanthamoeba* strains examined (Figures 2A,B), this demonstrated that the lehfilcon A material specifically allowed significantly lower aggregation versus all other materials tested. Visually, the no lens control and lehfilcon A showed individual trophozoites moving freely as individuals across the surface while other materials demonstrated aggregation to various degrees across the course of the experiment (Figures 2C,D; Supplementary Videos S1–S6). Supplementary Videos S1: ATCC 30010, S2: ATCC 30461, S3: ATCC 50370, S4: ATCC 50702, S5: ATCC 50703, S6: ATCC PRA-115.

Additionally, despite being seeded with the same number of cells in the same plate at the same time, the no lens control in most strains tested demonstrated a lower particle count in the field of view than all lenses tested. This was due to amoeba having a larger available space (flat-bottomed well vs. bowl of a contact lens), although they were consistently observed to be evenly dispersed throughout the well both in this study and in previous ones (Campolo et al., 2021). Further, it is a noticeable phenomenon that count increases in all lens materials and in all strains from time 0–0.5 h to 0.5–1.0 h. This is due not only to amoeba settling onto the lens, but also to their inclination to walk down to the bottom of the bowl of the lens upon adherence to the lens, thereby coming into the field of view and being counted. When observed, aggregation into spheroids is most often seen at the 0.5–1.0 h time point (demonstrated by the marked decrease in count), while non-aggregation results in consistently higher particle counts.

When examined individually (Figures 3–5; Supplementary Figures S3, S4), most strains demonstrated the similar trend of lehfilcon A maintaining a statistically higher cell count than other lens materials at most time points (*p* < 0.05). The exceptions to this were etafilcon A, which demonstrated little aggregation in ATCC 30010 or ATCC 50703. The ATCC 50703 strain was the only strain tested where cell count continued to increase over time for senofilcon A, samfilcon A, and omafilcon A. Senofilcon A produced the highest particle size (i.e., largest spheroid size) in ATCC 30010 and ATCC 30461, while fanfilcon A and/or comfilcon produced the largest spheroid size in ATCC 50370, ATCC 50703, and ATCC PRA-115, and omafilcon A produced the largest spheroid size in ATCC 50702. The particle size of the no lens control and lehfilcon A were not statistically different from each other at any time point in any strain tested. It is noted that some strain-material combinations produced a pronounced peak in spheroid size in later timepoints, which may be slightly reduced before 72 h (such as ATCC 50703 comfilcon A) or may continue to grow through the 72 h timepoint (such as ATCC 30461 comfilcon A), while the majority of other strain-material combinations demonstrated a more consistent particle size from at least 3.0–3.5 h onwards. To note, ATCC 50703 had the least stable spheroids of all strains, with several lens materials showing susbtantial early aggregation followed by deaggregation at later timepoints.

### Cell counts within aggregated spheroids

**Figure 6:**
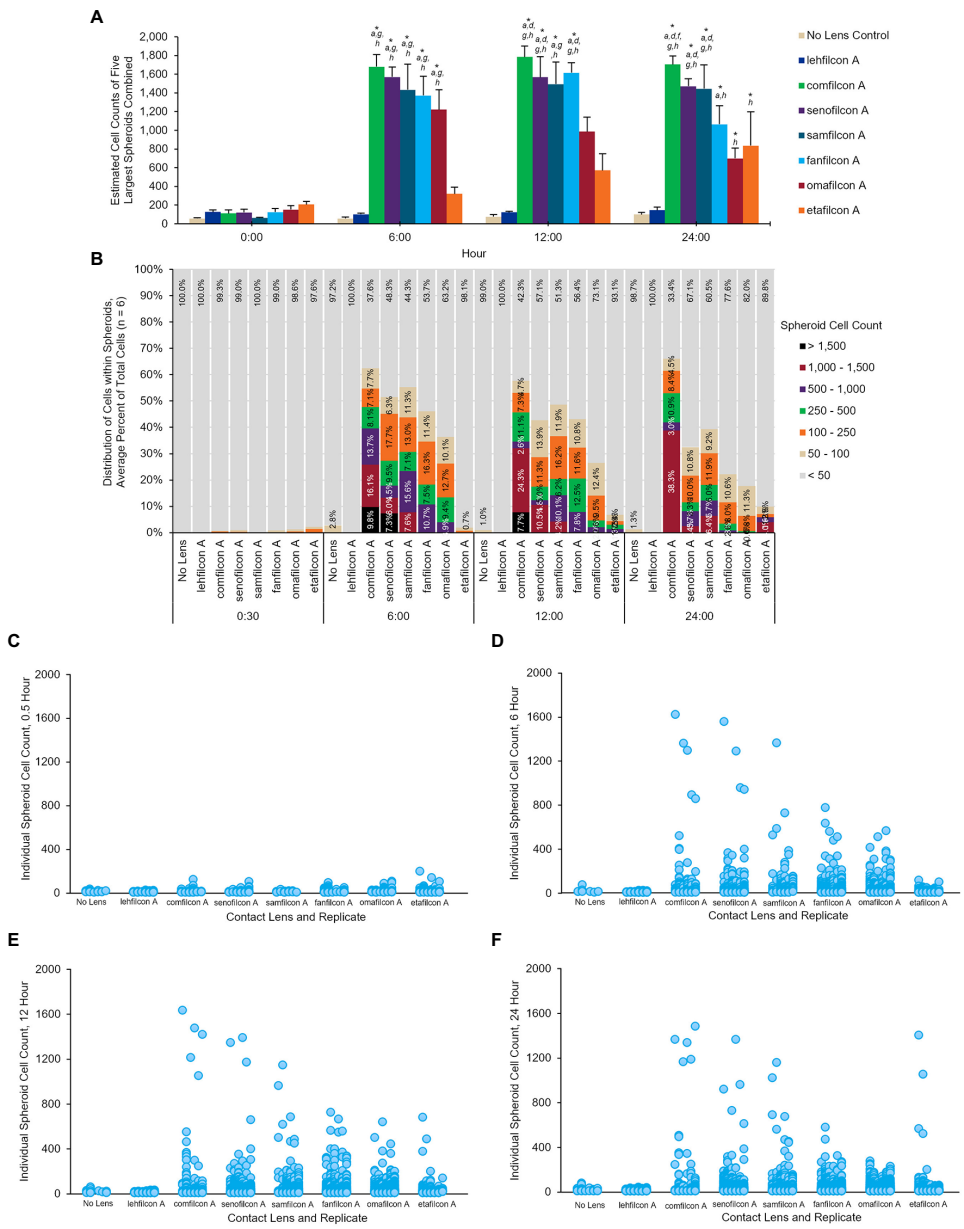
Once aggregated, cell counts of *Acanthamoeba polyphaga* (ATCC 30461) spheroids are maintained through 24 h. **(A)** Cell count of five largest spheroids on any one lens combined, calculated per lens type and presented at mean ± SE among 6 replicates. **(B)** Percentage of cells that are maintained in spheroids of various sizes over time, delineated by color for each spheroid size. Percentages are an average from 6 replicates per lens material. **(C–F)** Individual spheroid cell counts at the 0.5 h, 6 h, 12 h, and 24 h timepoints. Each individual spheroid on any lens, replicate, and timepoint is represented by a dot corresponding to its cell count. Replicates are visualized from left to right for each lens material (*n* = 6). Time 0 baseline is calculated from the 0.5 h to allow cells to adhere to the material. Analyzed *via* two-way repeat measure ANOVA. Within a given timepoint: (a) *p* < 0.05 vs. lehfilcon A, (b) *p* < 0.05 vs. comfilcon A, (c) *p* < 0.05 vs. senofilcon A, (d) *p* < 0.05 vs. omafilcon A, (e) *p* < 0.05 vs. samfilcon A, (f) *p* < 0.05 vs. fanfilcon A, (g) *p* < 0.05 vs. etafilcon A, (h) *p* < 0.05 vs. No Lens Control. Within a given lens type, *p < 0.05 baseline (0.5 h).

Each spheroid (defined as more than four cells touching at once) was analyzed to determine the number of cells it contained from baseline (0.5 h) through hour 24 (Figure 6). Each lens material was noted to have unique aggregation profiles: the No Lens Control and lehfilcon A maintained no significant aggregation through all 24 h. Etafilcon A did not demonstrate aggregation at hours 0.5, 6, or 12, but did have significantly more aggregation vs. its own baseline and vs. the No Lens Control at hour 24 (*p* < 0.05). Comfilcon A, senofilcon A, samfilcon A, and fanfilcon A maintained significantly larger spheroids than the No Lens Control and lehfilcon A at hours 6, 12, and 24. Omafilcon A showed signficant aggregation at hours 6 and 12, with a moderate dispersal of the spheroids by hour 24. Comfilcon A also demonstrated the largest spheroids by size (>1,500 cells per spheroid at multiple time points) while aggregating materials such as senofilcon A and samfilcon A were more likely to produce several spheroids of more moderate size (between 100 and 1,500 cells per spheroid).

### Visualization of encystment within *Acanthamoeba* spheroids

**Figure 7:**
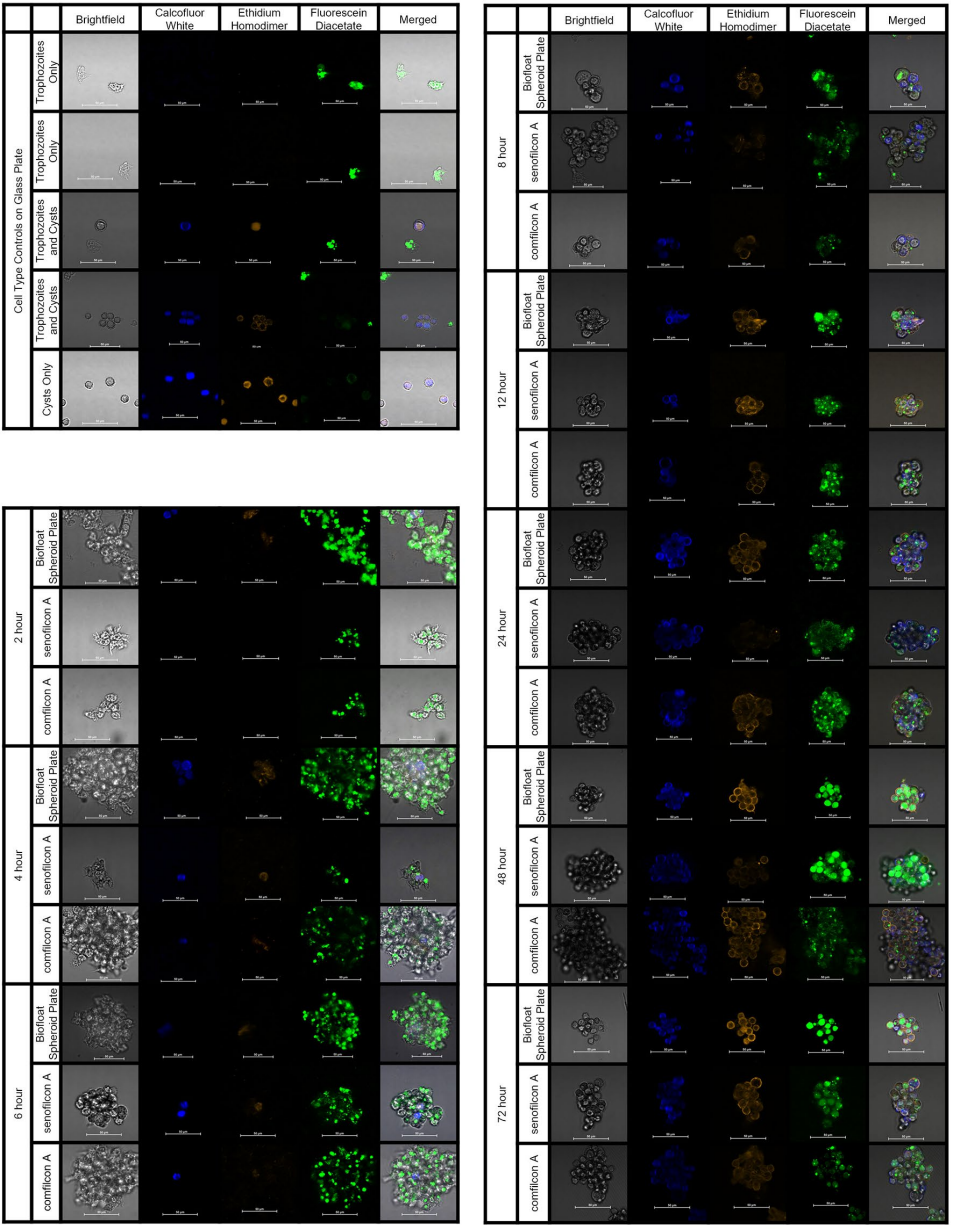
*Acanthamoeba polyphaga* (ATCC 30461) demonstrated encystment at 4 h on all three materials tested, and maintained encystment through 72 h. Controls: Control cells (trophozoites, and cysts pre-made *via* starvation) were imaged on a glass slide to indicate stain response prior to aggregation experiments. Aggregation: Representative images of fluorescently stained *Acanthmoeba* spheroids on a spheroid-producing Biofloat plate, senofilcon A, or comfilcon A, without other encystment-inducing substrates. Calcofluor white staining (DAPI filter, blue color) binds to the cellulose of cell walls and indicates cysts. Ethidium homodimer staining (TRITC filter, orange color) binds to nucleic acids and indicates compromised cells or cell death (able to be pentrated by stain and bind to nucleic acids) or presence of extracellular matrix. Fluoroscein diacetate (FITC filer, green color) is a dye that can penetrate trophozoite cell walls and indicates enzymatic activity. All scale bars equal 50 μm. Eight spheroids were created and imaged for each condition in separate wells and representative images were chosen at random.

To understand the formation of *Acanthamoeba* cysts within spheroids and evaluate if aggregation was a similar process regardless of material trigger, we utilized fluorescent confocal microscopy and three different spheroid-forming conditions: a BIOFLOAT™ spheroid plate, senofilcon A, and comfilcon A (Figure 7). Results were highly similar between all three materials tested, indicating that spheroids made on Biofloat spheroid plates were structurally similar to those made on contact lens materials. As early as 4 h, Calcofluor-white-positive cysts were observed on contact lens materials. Likewise, ethidium homodimer staining became evident in the vicinity of newly formed cysts, indicating the general building of an extracellular matrix, while still demonstrating some enzymatic activity *via* fluorescein diacetate staining. Notably, the ethidium homodimer staining outlining cell shapes (but not often filling a cell cytoplasm) indicates for the first time that *Acanthamoeba* spheroids may be forming an extracellular matrix as they age. Fluorescein diacetate was noted in spheroids at all timepoints and in both trophozoites and cysts, although it was more prominent in older spheroids, indicating mature spheroids are still viable, metabolically active, infectious cells (Garajová et al., 2019).

### Genomic analysis of *Acanthamoeba* on lens materials

**Figure 8:**
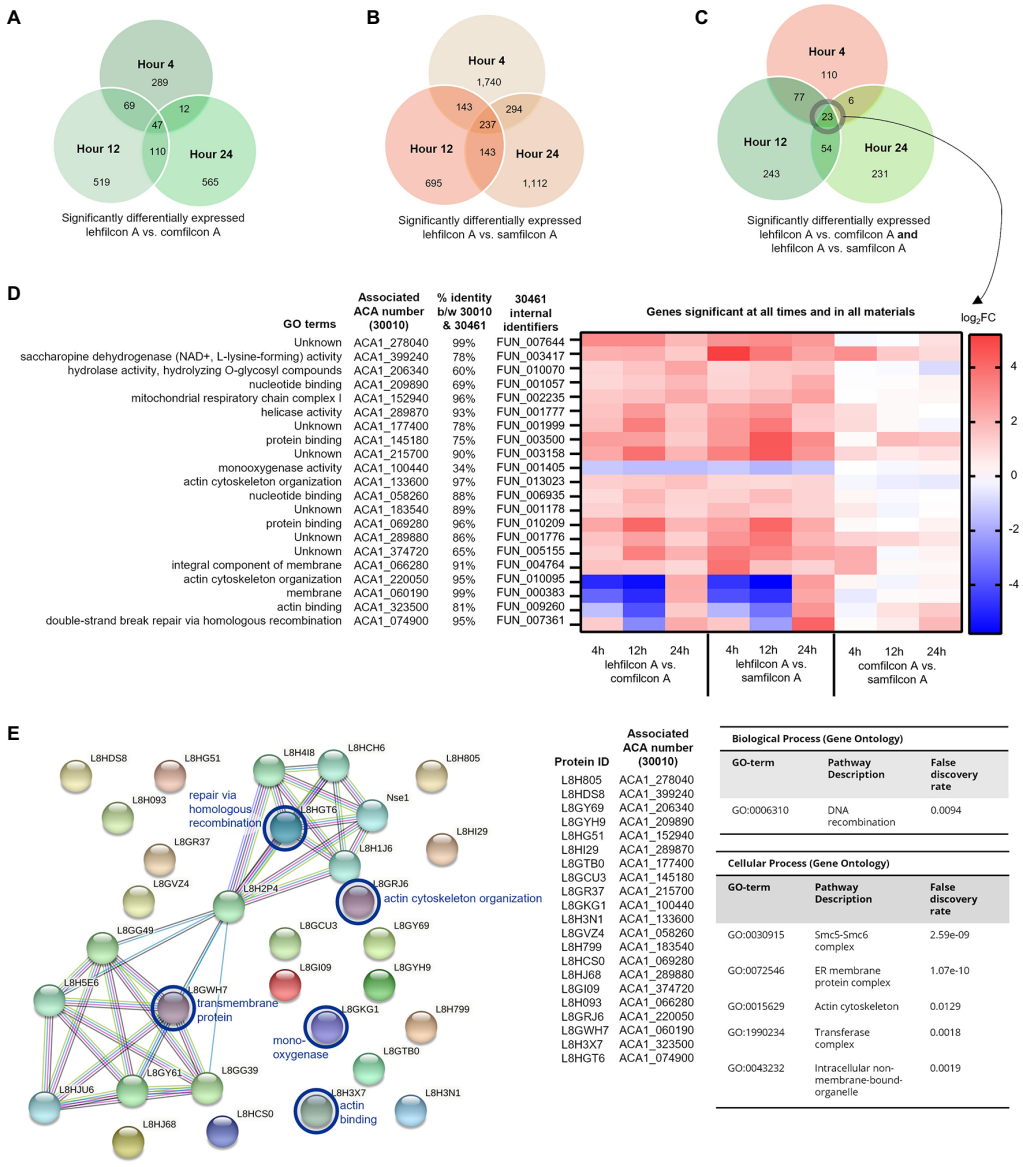
Genomic analysis of *Acanthamoeba polyphaga* (ATCC 30461) on three different contact lens materials at hours 4, 12, and 24. **(A)** Venn diagram of overlapping genes that were significantly different between lehfilcon A vs. comfilcon A, by hour, **(B)** Venn diagram of overlapping genes that were significantly different between lehfilcon A vs. samfilcon A, by hour, and **(C)** Venn diagram of overlapping genes that were significantly different between lehfilcon A vs. comfilcon A and lehfilcon A vs. samfilcon A, by hour. **(D)** 23 genes were significantly differentially expressed between lehfilcon A and both other materials, and in all three time points; *p* < 0.05, *n* = 6 per group. Genes are described according to locus identifier, closest identified homologue in ATCC 30010, and GO terms associated with ATCC 30010 protein according to AmoebaDB. **(E)** All 21 genes are visualized as proteins and their protein–protein interactions are identified *via* STRING: significant pathways are identified using a false discovery rate of <0.05. Proteins that were significantly upregulated in aggregating lenses at any timepoint are indicated in the protein–protein interaction map with a blue circle. All other proteins visualized were significantly downregulated in aggregating lenses compared to the non-aggregating lens. Further visualizations of other significant genes presented in Figures 9 and 10.

**Figure 9:**
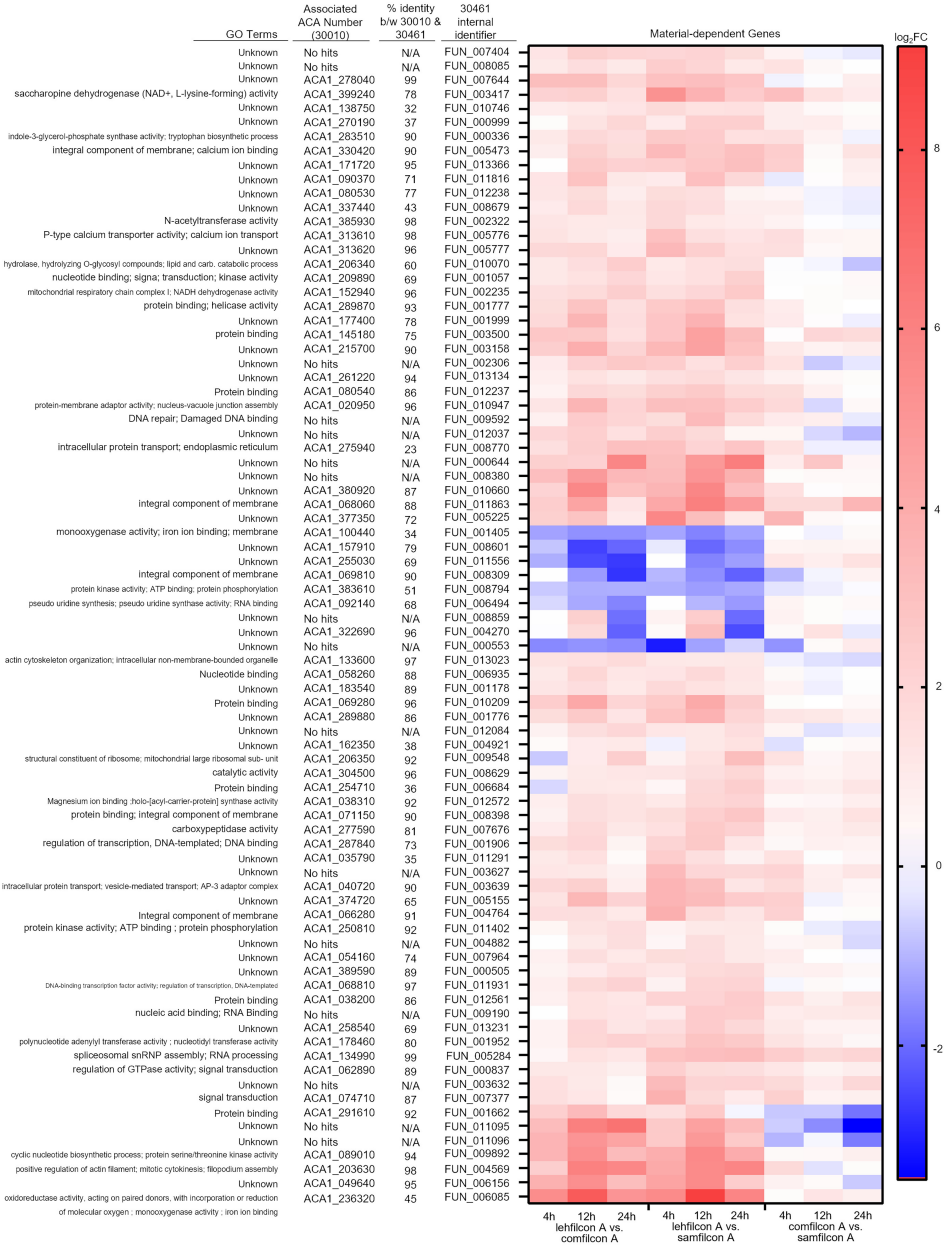
Genomic analysis of *Acanthamoeba polyphaga* (ATCC 30461) on three different contact lens materials at hours 4, 12, and 24. Eighty two genes were found to be significantly differentially regulated in at least two consecutive time points; *p* < 0.05, *n* = 6 per group. Genes were clustered according to gene expression pattern with those depicted falling into a predominantly contact lens material-dependent expression pattern. Heat maps display the kinetics of log2 fold change in expression on lehfilcon A relative to comfilcon A (left), lehfilcon A relative to samfilcon A (middle), and comfilcon A relative to samfilcon A (right). Genes are described according to locus identifier, closest identified homologue in ATCC 30010, and GO terms associated with ATCC 30010 protein according to AmoebaDB.

**Figure 10:**
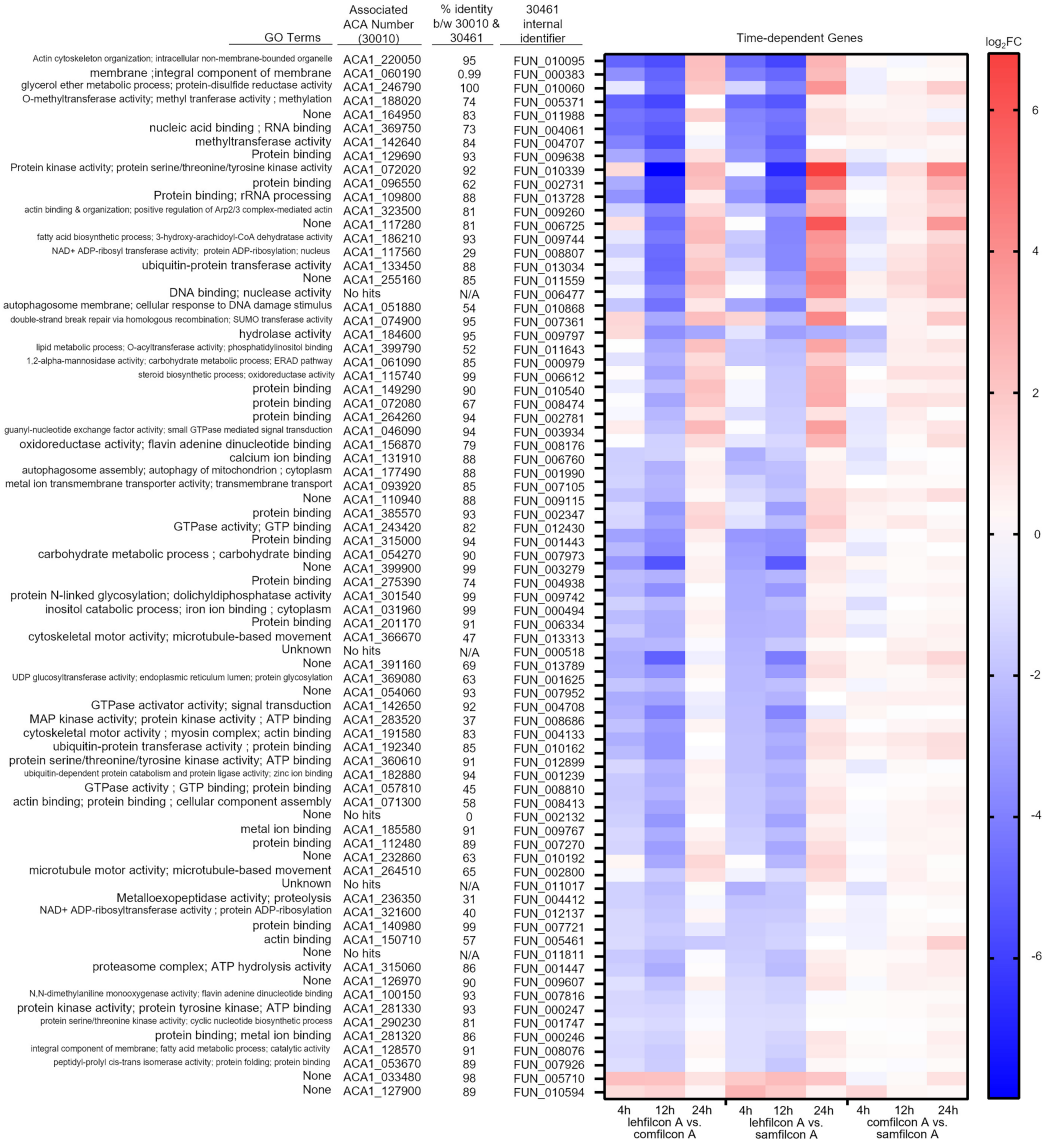
Genomic analysis of *Acanthamoeba polyphaga* (ATCC 30461) on three different contact lens materials at hours 4, 12, and 24. Seventy seven genes were found to be significantly differentially regulated in at least two consecutive time points; *p* < 0.05, *n* = 6 per group. Genes were clustered according to gene expression pattern with those depicted falling into a time and material-dependent expression pattern. Heat maps display the kinetics of log2 fold change in expression on lehfilcon A relative to comfilcon A (left), lehfilcon A relative to samfilcon A (middle), and comfilcon A relative to samfilcon A (right). Genes are described according to locus identifier, closest identified homologue in ATCC 30010, and GO terms associated with ATCC 30010 protein according to AmoebaDB.

Following the observation of material-dependent *Acanthamoeba* behavior leading to either independently motile trophozoites or enmeshed spheroids, we analyzed the transcriptome of *Acanthamoeba* ATCC 30461 (a commonly utilized strain) on lehfilcon A, comfilcon A, samfilcon A, and the no lens control (Figures 8–10, online repository for complete data set). This study was undertaken to identify which genes or potential pathways may be contributing to the aggregation or may be contributing to the persistence of a spheroid (when it does not dissociate over time), and downstream cellular changes in *Acanthamoeba* as a result of being part of a spheroid. Lehfilcon A was again consistently noted as a non-aggregating lens. While both comfilcon A and samfilcon A dependably demonstrated aggregation, more significantly differentially expressed genes were found at all time points in lehfilcon A vs. samfilcon A than lehfilcon A vs. comfilcon A.

Twenty three genes were significantly differentially expressed at all timepoints (4, 12 and 24 h) in lehfilcon A vs. comfilcon A and samfilcon A, including two tRNA genes that were removed from the detailed analysis. The resulting 21 genes were visualized *via* heatmap and the homologous genes from ATCC 30010 (Neff strain) were identified (Figure 8D). All 21 genes demonstrated some degree of homology with ATCC 30010, and the majority demonstrated over 75% homology, although not all genes have inferred functions; 6 of the 21 genes are currently unknown. Further, all 21 genes possessed a similar differential expression profile between lehfilcon A vs. comfilcon A and lehfilcon A vs. samfilcon A (that is, if a gene was downregulated in one, it was downregulated in the other, and so on). Overall, as indicated by the white coloration in the heatmap, the genes that were significantly differentially expressed between the aggregating lenses and the non-aggregating lens demonstrated very little difference when the two aggregating lenses were compared to each other, thus further signifying that these genes are involved in highly similar behavioral responses to lens materials. Protein–protein interactions and the significantly differentially expressed pathways were further identified (Figure 8E) and alterations were noted in pathways involving the actin cytoskeleton, intracellular vesicle formation, and metabolic activity. Further visualizations along with Neff strain homology and GO term descriptions can be found in Figures 9, 10.

### Aggregated *Acanthamoeba* impedes disinfection efficacy

**Figure 11:**
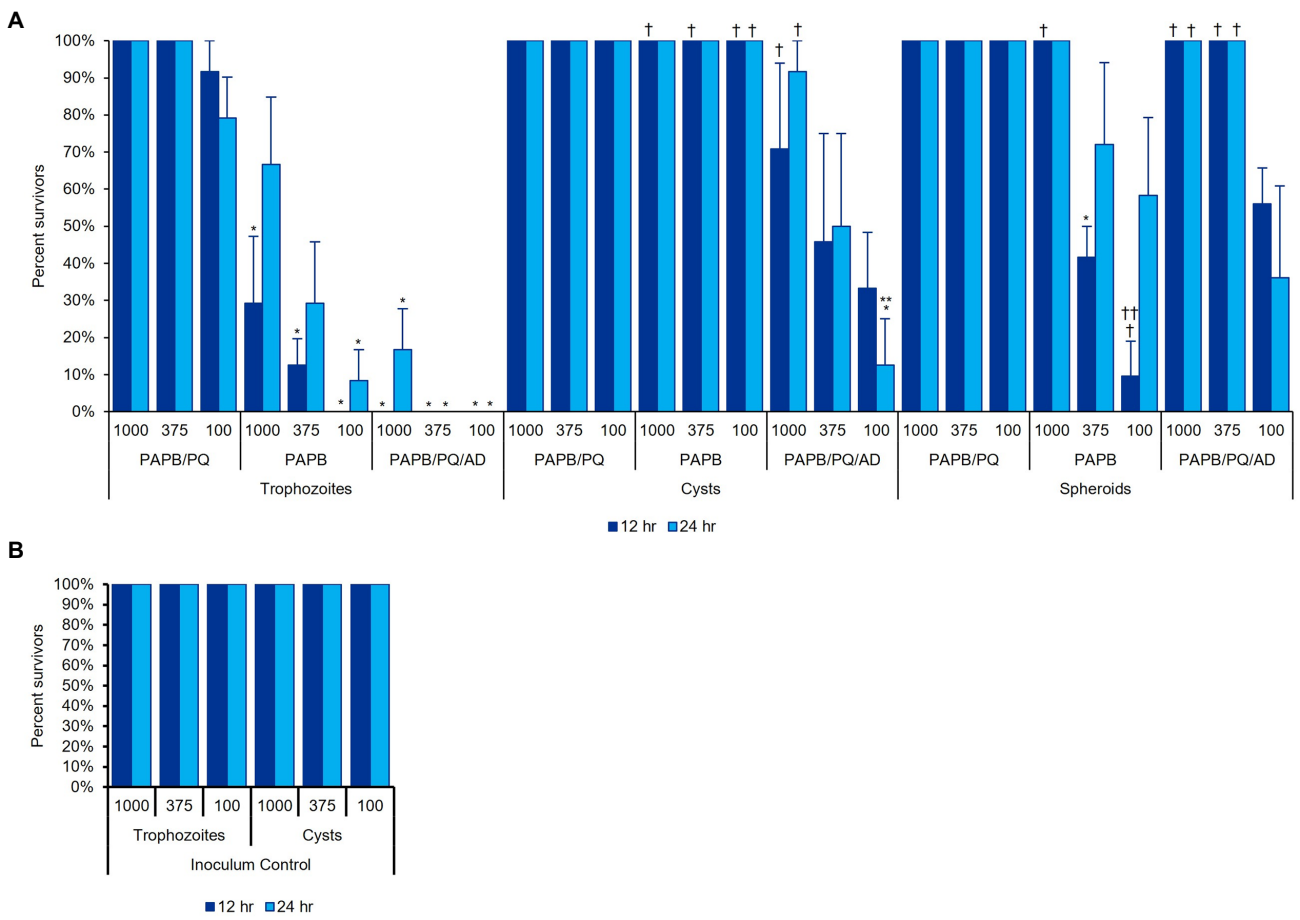
*Acanthamoeba polyphaga* (ATCC 30461) in three different cell concentrations (1,000, 375, or 100 cells per 100 μl) was used to examine MPS disinfection efficacy against **(A)** trophozoites, cysts, and spheroids, after cells had adhered to the plate for 12 or 24 h. **(B)** An inoculum control was run concurrently with the same cultures used in **(A)**. Each bar represents a mean ± SE of 3 replicates, each replicate being composed of 8 wells from which percent survivorship was calculated. Statistical analysis *via* 2-way ANOVA: comparisons versus a MPS within the same cell condition and same time, *p < 0.05 vs. PAPB/PQ, **p < 0.05 vs. PAPB; comparisons versus a cell condition within the same MPS and same time, ^†^*p* < 0.05 vs. trophozoites, ^††^*p* < 0.05 vs. cysts.

To determine MPS disinfection efficacy of *Acanthamoeba* in different cellular formations (Figure 7), *Acanthamoeba* were assessed for biocide resistance in either trophozoite, cyst, or spheroid forms after cells had been allowed to adhere (or aggregate) in plates for 12 or 24 h (Figure 11). Consistent with published findings (Gabriel et al., 2019; Walters et al., 2022), cells in the trophozoite form were the most susceptible to MPS biocides while cysts were significantly more challenging to disinfect. PAPB/PQ showed little ability to kill any form, with 100% survival for spheroids and cysts across all cell concentrations. Likewise, PAPB had no ability to disinfect cysts though some efficacy was observed against trophozoites. The increased survivability for trophozoites with PAPB over time may be due to individual cysts forming by 24 h that PAPB has no ability to kill. PAPB demonstrated less efficacy against spheroids compared to individual trophozoites at all concentrations and time periods despite the cells originating from the same stock. PAPB/PQ/AD demonstrated nearly complete kill of trophozoites for all concentrations and time points with limited survivors at the highest concentration of trophozoites at 24 h, again likely due to the development of individual cysts within the population. PAPB/PQ/AD also had the best efficacy against cysts due to the activity of alexidine where only 50% of the 375 cysts/well condition survived. In contrast, 100% of the spheroid wells survived disinfection with PAPB/PQ/AD for both timepoints at the two highest cell concentrations, and 50% spheroid survival at the lowest concentration, highlighting how the physical barrier of aggregated cells and subpopulations of cysts presented a substantial challenge for MPS disinfection compared to individual trophozoites and cysts at the same concentration.

## Discussion

*Acanthamoeba* and its risk to contact lens users remains at the forefront of ophthalmology and optometry due the devastating consequences of infections and limited options for treatment (Siddiqui and Khan, 2012; Szentmary et al., 2019). While studied for decades, *Acanthamoeba* research has only recently expanded to utilize robust -omic methods to understand more about this amoeba’s dynamic lifecycle (Bernard et al., 2022). Even now, limited genome annotation and no stable methods for impacting gene expression have significantly hampered *Acanthamoeba* research where the pathogenesis of other ocular microorganisms like *Pseudomonas* are well-described and the risk factors for infection clearly delineated (Gu et al., 2022). Equally, the infrequent diagnosis of *Acanthamoeba* keratitis’ has prevented significant investment in treatment development and infection prevention, despite devastating outcomes for those affected (Siddiqui and Khan, 2012; Szentmary et al., 2019). Most outreach and publicity stems from survivors, though education of contact lens users and, equally, their optometrists, is being pioneered by experts around the globe. Currently, the sole defense for contact lens users beyond water avoidance is the disinfecting solutions utilized to clean contact lenses (Arshad et al., 2019, 2021; British Contact Lens Association, 2021). Most disinfecting solutions were developed to reduce the bacterial and lipid/protein deposit load on a lens as opposed to engineering a lens or lens solution with a more challenging organism like *Acanthamoeba* in mind (although, the reduction of bacteria binding to a lens could limit the nutrient source and proliferation for *Acanthamoeba*). The regulatory requirements for *Acanthamoeba* multi-purpose solution disinfection efficacy continue to lag despite calls for action (Jobson Medical Information, LLC, 2009; Primary Care Optometry News, 2014). However, no multi-purpose solutions have a requirement to disinfect *Acanthamoeba* (ISO 14729:2001/A1:2010, 2010) and effective chemicals available against *Acanthamoeba* are unable to be utilized at sufficient concentrations due to toxicity to the cornea. Equally, the most effective products against all types of *Acanthamoeba* are hydrogen peroxide based (Walters et al., 2022). However, many consumers avoid hydrogen peroxide use because of the risk of accidently burning the corneal surface through misuse, despite the disinfection benefits, as well as the slightly more complicated user instructions. Here, we have shown it is most important to keep *Acanthamoeba* in their most susceptive state (trophozoites) where most multi-purpose solutions offer some level of disinfecting ability.

Contact lenses have evolved significantly from the polymethyl methacrylate and original rigid gas permeable lenses. Contact lens manufacturers must continue to evolve new materials to increase oxygen permeability and wettability to combat user discomfort that can cause patients to revert to glasses. For *Acanthamoeba* keratitis with small outbreaks associated with poor MPS efficacy, little examination has been done to evaluate the role of the contact lens in *Acanthamoeba* pathogenesis. Here, we have described a novel finding that many strains of *Acanthamoeba* will aggregate in response to specific contact lens materials. This is a significant potential risk to patients as *Acanthamoeba* aggregation is a precursor to encystment (Coulon et al., 2010; Bernard et al., 2022). As part of its natural life cycle, *Acanthamoeba* will aggregate into small clusters to encyst. Likely a protective measure, aggregation in response to a newly toxic environment makes evolutionary sense where even if the entire population fails to encyst, at least some individuals, potentially at the center of an spheroid, may survive the unfavorable environment (Coulon et al., 2010). To our knowledge, this is the first time this aggregation and encystment phenomenon has been observed in this short duration where issues like food availability and chemical induction were not at play. Trophozoites were induced to aggregate and encyst by the material they were in contact with, versus a change in chemical or nutrient availability commonly used in other studies (Coulon et al., 2010, 2012; Bernard et al., 2022). Gene expression evaluation indicated alterations in encystment pathways where actin cytoskeleton rearrangement is critical to the aggregation pathways as well as changes in metabolic activity and intracellular vesicles. Fluorescent confocal microscopy confirmed the presence of cysts as early as four hours after introduction to a contact lens surface. This demonstrates a significantly faster aggregation than has been observed in chemical induction such as Neff’s encystment media (>24 h) or starvation (7–14 days; Aqeel et al., 2013). The contact lens surface demonstrated such a significant risk that *Acanthamoeba* actively initiated a terminal differentiation resulting in cysts far earlier than normal nutrient unavailability would stimulate. Other contact lens-associated risks were apparent as well, including the upregulation of autophagy and ubiquitination in the aggregating lenses verses lehfilcon A. Interestingly, spheroids removed from contact lens materials can de-aggregate on polystyrene or lehfilcon A though cysts within the spheroid remain cysts without a food source (Supplementary Video S7). For polystyrene controls and lehfilcon A, there was no indication that any binary fission occurred based on cell count, and eventually individual trophozoites encysted though active trophozoites were visible through the 72 h despite no nutrient source. This supports previous work that shows trophozoites will maintain motility through 24 h with no decrease in activity even without nutrients (Campolo et al., 2021). Here, *Acanthamoeba* trophozoites show a remarkable response to a surface they identified as inhospitable, responding quickly (<1 h) to many contact lens materials, aggregating and initiating encystment (< 4 h) despite no chemical induction beyond the contact lens surface. No nutrients are provided on the lens but this is not a long enough time period to be considered starvation. The properties of the aggregation-inducing contact lens materials that trigger this response are unknown and would require future investigation, but this response could potentially be due to diminished water content or surface topography of the contact lens materials. *Acanthamoeba* prefers to exist at the interfaces between soil and water, and surfaces indicating a strong water-aversion (Liang et al., 2022; Wesley et al., 2022) may trigger the protective response.

The risk of aggregation and encystment for *Acanthamoeba* and its potential to cause human disease has so far been dependent on the ability to kill *Acanthamoeba* before patient contact. For contact lenses, the creation of spheroids and cysts could significantly hamper multi-purpose solutions from adequately disinfecting *Acanthamoeba* from lenses. Here, we demonstrated that spheroids can resist disinfection in clinically relevant, low cell concentrations (Li et al., 2020). Seeding a well at a concentration of ~100 trophozoites/well was equivalent to a low inoculum concentration of 5.0×10^2^ cells/mL. Most microbial efficacy standards require inoculation at 10^5^–10^6^ cells/mL for a microorganism, and a resulting 3-log disinfection efficacy for the solution being tested (ISO 14729:2001/A1:2010, 2010). Here, we demonstrated that spheroids could survive disinfection at far lower densities than any standard is currently evaluating. The resistance of the spheroid is two-fold: the spheroid itself provides a protective layer preventing penetration of biocides to interior cells, and the rapid formation of cysts at the center of spheroids offers cells with a naturally high biocide resistance. Interestingly, even biocides like alexidine known to be capable of killing cysts were less effective against spheroids despite showing efficacy against individual trophozoites and cysts.

This data indicates the need for continued research into *Acanthamoeba’s* interactions with contact lenses. For instance, the timeline of encystment noted in the observations here, as amoeba appear to encyst on contact lenses much faster than they do *via* starvation or *via* encystment media, should be further studied as the transcriptome analysis did not show differentially expressed genes from known encystment genes (Dudley et al., 2009; Rolland et al., 2020; Bernard et al., 2022). We attribute this to the speed at which encystment is being induced which is significantly faster than other studies that used chemical induction of encystment. Further, the observations made here regarding disinfection efficacy should be followed up with in *in vivo* examinations to determine the risk that aggregating lenses may pose to patients. Similarly, to our knowledge, there are no meta-analyses relating contact lenses themselves to *Acanthamoeba* keratitis cases or prevalence, and this would be a critical investigation to supplement the information presented here. Finally, while we did observe variability between strains, with ATCC 50703 being the most divergent from the group, we do note that when all strains are combined the results are consistently statistically significant regarding which lenses do and do not promote aggregation. We thought it important to show the differences between genotypes even when some strains did not show the strong aggregation behaviors found in others. These divergent strains or behaviors merit future investigation.

Together, this study demonstrates that *Acanthamoeba* behavior can be significantly altered by different polymeric surface properties, particularly those found in contact lens materials. That behavior, which results in a protective mechanism that promotes *Acanthamoeba* encystment far faster than natural stressors like starvation, may contribute to the pathogenesis of this organism by making it resistant to available disinfection methods. *Acanthamoeba* spheroids and the underlying surface properties that lead to their formation represent an under-investigated field of research. While promotion of proper disinfection of lenses is critical to patient safety, now it becomes equally important to impress on patients that their contact lenses should never come in contact with any water source that may contain *Acanthamoeba*. With contact lens materials continuing to diversify, *Acanthamoeba’s* response to contact lenses must be further studied to understand the complete implications to patient safety.

## Data availability statement

The data presented in the study are deposited in the NCBI BioProject repository, accession numbers PRJNA903937 (https://www.ncbi.nlm.nih.gov/bioproject/PRJNA903937) and PRJNA905484 (https://www.ncbi.nlm.nih.gov/bioproject/PRJNA905484).

## Author contributions

AC, RP, RW, JK, CR, PS, BP, and MC were involved in conceptualization. AC, RP, RW, MT, EM, VH, JK, BP, and MC were involved in data curation and methodology. AC, RP, CR, BP, and MC were responsible for formal analysis. AC, RP, VH, JK, BP, and MC were involved in validation. AC, RP, RW, MT, EM, CR, BP, and MC conducted visualization. PS and MC were responsible for project administration, resources, and supervision. AC and MC conducted writing of the original draft. All authors participated in manuscript review and editing. All authors had full access to all the data in the study and had final responsibility for the decision to submit for publication. All authors contributed to the article and approved the submitted version.

## Funding

This research was funded by Alcon, which provided all financial support. CR reports grants from Purdue University, outside of the scope of the submitted work.

## Conflict of interest

The funder was not involved in the study design, collection, analysis, interpretation of data, or the writing of this article. The funder approved the decision to submit for publication. All authors except CR are employees of Alcon Research.

The remaining author declares that the research was conducted in the absence of any commercial or financial relationships that could be construed as a potential conflict of interest.

## Publisher’s note

All claims expressed in this article are solely those of the authors and do not necessarily represent those of their affiliated organizations, or those of the publisher, the editors and the reviewers. Any product that may be evaluated in this article, or claim that may be made by its manufacturer, is not guaranteed or endorsed by the publisher.

## Supplementary material

The Supplementary material for this article can be found online at: https://www.frontiersin.org/articles/10.3389/fmicb.2022.1089092/full#supplementary-material

## References

[ref1] *Acanthamoeba* polyphaga (Pushkarew). (2019). Encyclopedia of life. Available at: https://eol.org/pages/593787 (Accessed October 6, 2022).

[ref2] Ahearn D. G. Simmons R. B. Ward M. A. Stulting R. D. (2012). Potential resistant Morphotypes of *Acanthamoeba castellanii* expressed in multipurpose contact lens disinfection systems. Eye Contact Lens 38, 400–405. doi: 10.1097/ICL.0b013e318261ab1f, PMID: 22858984

[ref3] Antonelli A. Favuzza E. Galano A. Montalbano Di Filippo M. Ciccone N. Berrilli F.. (2018). Regional spread of contact lens-related *Acanthamoeba* keratitis in Italy. New Microbiol. 41, 83–85. PMID: 29505068

[ref4] Aqeel Y. Siddiqui R. Iftikhar H. Khan N. A. (2013). The effect of different environmental conditions on the encystation of *Acanthamoeba* castellanii belonging to the T4 genotype. Exp. Parasitol. 135, 30–35. doi: 10.1016/j.exppara.2013.05.017, PMID: 23769934

[ref5] Arshad M. Carnt N. Tan J. Ekkeshis I. Stapleton F. (2019). Water exposure and the risk of contact lens-related disease. Cornea 38, 791–797. doi: 10.1097/ICO.0000000000001898, PMID: 30789440

[ref6] Arshad M. Carnt N. Tan J. Stapleton F. (2021). Compliance behaviour change in contact lens wearers: a randomised controlled trial. Eye (Lond.) 35, 988–995. doi: 10.1038/s41433-020-1015-9, PMID: 32546749 PMC8027657

[ref7] bcl-convert (2021). A proprietary Illumina software for the conversion of bcl files to basecalls. https://support-docs.illumina.com/SW/BCL_Convert/Content/SW/FrontPages/BCL_Convert.htm (Accessed November 30, 2022).

[ref8] Beattie T. K. Tomlinson A. Seal D. V. McFadyen A. K. (2011). Salicylate inhibition of acanthamoebal attachment to contact lenses. Optom. Vis. Sci. 88, 1422–1432. doi: 10.1097/OPX.0b013e318230f574, PMID: 21926650

[ref9] Bernard C. Locard-Paulet M. Noël C. Duchateau M. Giai Gianetto Q. Moumen B.. (2022). A time-resolved multi-omics atlas of *Acanthamoeba castellanii* encystment. Nat. Commun. 13:4104. doi: 10.1038/s41467-022-31832-0, PMID: 35835784 PMC9283445

[ref10] British Contact Lens Association (2021). Raising public awareness of no water and *Acanthamoeba* keratitis. Available at: https://anchor.fm/bcla/episodes/Raising-public-awareness-of-nowater-and-AK-es7l9b/ (Accessed February 4, 2022).

[ref11] Brown A. C. Ross J. Jones D. B. Collier S. A. Ayers T. L. Hoekstra R. M.. (2018). Risk factors for *Acanthamoeba* keratitis—a multistate case–control study, 2008–2011. Eye Contact Lens 44, S173–S178. doi: 10.1097/ICL.0000000000000365 28099282

[ref12] Buck S. L. Rosenthal R. A. (1996). A quantitative method to evaluate neutralizer toxicity against *Acanthamoeba castellanii*. Appl. Environ. Microbiol. 62, 3521–3526. doi: 10.1128/aem.62.9.3521-3526.1996, PMID: 8795247 PMC168153

[ref13] Campolo A. Harris V. Walters R. Miller E. Patterson B. Crary M. (2021). Continuous real-time motility analysis of *Acanthamoeba* reveals sustained movement in absence of nutrients. Pathogens 10:995. doi: 10.3390/pathogens10080995, PMID: 34451459 PMC8398851

[ref14] Carnt N. Hoffman J. M. Verma S. Hau S. Radford C. F. Minassian D. C.. (2018). *Acanthamoeba* keratitis: confirmation of the UK outbreak and a prospective case-control study identifying contributing risk factors. Br. J. Ophthalmol. 102, 1621–1628. doi: 10.1136/bjophthalmol-2018-312544, PMID: 30232172

[ref15] Carnt N. A. Subedi D. Connor S. Kilvington S. (2020). The relationship between environmental sources and the susceptibility of *Acanthamoeba* keratitis in the United Kingdom. PLoS One 15:e0229681. doi: 10.1371/journal.pone.0229681, PMID: 32160218 PMC7065798

[ref16] Chelkha N. Jardot P. Moussaoui I. Levasseur A. La Scola B. Colson P. (2020). Core gene-based molecular detection and identification of *Acanthamoeba* species. Sci. Rep. 10:1583. doi: 10.1038/s41598-020-57998-5, PMID: 32005846 PMC6994504

[ref17] Coulon C. Collignon A. McDonnell G. Thomas V. (2010). Resistance of *Acanthamoeba* cysts to disinfection treatments used in health care settings. J. Clin. Microbiol. 48, 2689–2697. doi: 10.1128/JCM.00309-10, PMID: 20519477 PMC2916629

[ref18] Coulon C. Dechamps N. Meylheuc T. Collignon A. McDonnell G. Thomas V. (2012). The effect of in vitro growth conditions on the resistance of *Acanthamoeba* cysts. J. Eukaryot. Microbiol. 59, 198–205. doi: 10.1111/j.1550-7408.2012.00612.x, PMID: 22353167

[ref19] Cruz A. R. S. Rivera W. L. (2014). Genotype analysis of *Acanthamoeba* isolated from human nasal swabs in the Philippines. Asian Pac. J. Trop. Med. 7, S74–S78. doi: 10.1016/S1995-7645(14)60206-6, PMID: 25312195

[ref20] de Lacerda A. G. Lira M. (2021). *Acanthamoeba* keratitis: a review of biology, pathophysiology and epidemiology. Ophthalmic Physiol. Opt. 41, 116–135. doi: 10.1111/opo.12752, PMID: 33119189

[ref21] Dobin A. Davis C. A. Schlesinger F. Drenkow J. Zaleski C. Jha S.. (2012). STAR: ultrafast universal RNA-seq aligner. Bioinformatics 29, 15–21. doi: 10.1093/bioinformatics/bts635, PMID: 23104886 PMC3530905

[ref22] Douglas M. (1930). Notes on the calssification of the amoeba found by Castellani in cultures of a yeast-like fungus. J. Trop. Med. Lond. 33, 258–259.

[ref23] Dudley R. Jarroll E. L. Khan N. A. (2009). Carbohydrate analysis of *Acanthamoeba castellanii*. Exp. Parasitol. 122, 338–343. doi: 10.1016/j.exppara.2009.04.009 19389397

[ref24] Gabriel M. M. McAnally C. Bartell J. Walters R. Clark L. Crary M.. (2019). Biocidal efficacy of a hydrogen peroxide lens care solution incorporating a novel wetting agent. Eye Contact Lens 45, 164–170. doi: 10.1097/ICL.0000000000000549, PMID: 30138250

[ref25] Garajová M. Mrva M. Vaškovicová N. Martinka M. Melicherová J. Valigurová A. (2019). Cellulose fibrils formation and organisation of cytoskeleton during encystment are essential for *Acanthamoeba* cyst wall architecture. Sci. Rep. 9:4466. doi: 10.1038/s41598-019-41084-6, PMID: 30872791 PMC6418277

[ref26] Gast R. J. (2001). Development of an *Acanthamoeba-*specific reverse dot-blot and the discovery of a new ribotype. J. Eukaryot. Microbiol. 48, 609–615. doi: 10.1111/j.1550-7408.2001.tb00199.x, PMID: 11831768

[ref27] Gatti S. Cevini C. Bruno A. Penso G. Rama P. Scaglia M. (1998). In vitro effectiveness of povidone-iodine on *Acanthamoeba i*solates from human cornea. Antimicrob. Agents Chemother. 42, 2232–2234. doi: 10.1128/AAC.42.9.2232, PMID: 9736540 PMC105790

[ref28] Griffiths A. J. (1969). “Encystment in amoebae” in Advances in Microbial Physiology. eds. Rose A. H. Wilkinson J. F. (Cambridge, MA: Academic Press), 105–129.

[ref29] Gu X. Lu X. Lin S. Shi X. Shen Y. Lu Q.. (2022). A comparative genomic approach to determine the virulence factors and horizontal gene transfer events of clinical *Acanthamoeba* isolates. Microbiol. Spectr. 10:e0002522. doi: 10.1128/spectrum.00025-22, PMID: 35416714 PMC9045148

[ref30] Gurevich A. Saveliev V. Vyahhi N. Tesler G. (2013). QUAST: quality assessment tool for genome assemblies. Bioinformatics 29, 1072–1075. doi: 10.1093/bioinformatics/btt086, PMID: 23422339 PMC3624806

[ref31] Ibrahim Y. W. Boase D. L. Cree I. A. (2009). How could contact lens wearers be at risk of *Acanthamoeba* infection? A Review. J. Optom. 2, 6–60. doi: 10.3921/joptom.2009.60

[ref32] ISO 14729:2001/A1:2010 (2010). Ophthalmic Optics–Contact Lens Care Products–Microbiological Requirements and TestMethods for Products and Rgimens for HygienicManagement of Contact Lenses; International Organization for Standardization: Geneva, Switzerland.

[ref34] John T. Desai D. Sahm D. (1989). Adherence of *Acanthamoeba castellanii* cysts and trophozoites to unworn soft contact lenses. Am. J. Ophthalmol. 108, 658–664. doi: 10.1016/0002-9394(89)90857-X, PMID: 2596545

[ref35] Johnston S. P. Sriram R. Qvarnstrom Y. Roy S. Verani J. Yoder J.. (2009). Resistance of *Acanthamoeba c*ysts to disinfection in multiple contact lens solutions. J. Clin. Microbiol. 47, 2040–2045. doi: 10.1128/JCM.00575-09, PMID: 19403771 PMC2708465

[ref76] Jon P. Jason S. (2019). nextgenusfs/funannotate: funannotate v1.5.3 (1.5.3). Zenodo. doi: 10.5281/zenodo.2604804, PMID: 19910308

[ref36] Kilvington S. (1993). *Acanthamoeba* trophozoite and cyst adherence to four types of soft contact lens and removal by cleaning agents. Eye 7, 535–538. doi: 10.1038/eye.1993.116, PMID: 8253233

[ref37] Kilvington S. Heaselgrave W. Powell H. Lally J. (2009). Physiological response of *Acanthamoeba* trophozoites to multipurpose contact lens solutions: aggregation and resistance to disinfection. Invest. Ophthalmol. Vis. Sci. 50:6360.

[ref38] Kilvington S. Lam A. (2013). Development of standardized methods for assessing biocidal efficacy of contact lens care solutions against *Acanthamoeba* trophozoites and cysts. Invest. Ophthalmol. Vis. Sci. 54, 4527–4537. doi: 10.1167/iovs.13-11927, PMID: 23745008

[ref39] Kilvington S. Larkin D. F. (1990). *Acanthamoeba* adherence to contact lenses and removal by cleaning agents. Eye (Lond.) 4, 589–593. doi: 10.1038/eye.1990.82, PMID: 2226989

[ref40] Lee S. M. Lee J. E. Lee D. I. Yu H. S. (2018). Adhesion of *Acanthamoeba* on cosmetic contact lenses. J. Korean Med. Sci. 33:e26. doi: 10.3346/jkms.2018.33.e26 29318793 PMC5760811

[ref41] Lee G.-H. Lee J.-E. Park M.-K. Yu H.-S. (2016). Adhesion of *Acanthamoeba* on silicone hydrogel contact lenses. Cornea 35, 663–668. doi: 10.1097/ICO.0000000000000788, PMID: 26938330

[ref42] Li S. Bian J. Wang Y. Wang S. Wang X. Shi W. (2020). Clinical features and serial changes of *Acanthamoeba* keratitis: an in vivo confocal microscopy study. Eye 34, 327–334. doi: 10.1038/s41433-019-0482-3, PMID: 31292523 PMC7002551

[ref43] Li B. Dewey C. N. (2011). RSEM: accurate transcript quantification from RNA-Seq data with or without a reference genome. BMC Bioinformatics 12:323. doi: 10.1186/1471-2105-12-323, PMID: 21816040 PMC3163565

[ref44] Liang S. Shows A. Dunbar D. Sharma V. Shi C. X. Wu J. (2022). Antifouling studies of Lehfilcon a silicone hydrogel contact lens. Invest. Ophthalmol. Vis. Sci. 63:532-A0230

[ref45] Lin Y. Yuan J. Kolmogorov M. Shen M. W. Chaisson M. Pevzner P. A. (2016). Assembly of long error-prone reads using de Bruijn graphs. Proc. Natl. Acad. Sci. U. S. A. 113, E8396–E8405. doi: 10.1073/pnas.1604560113, PMID: 27956617 PMC5206522

[ref46] Lloyd D. (2014). Encystment in *Acanthamoeba castellanii*: a review. Exp. Parasitol. 145, S20–S27. doi: 10.1016/j.exppara.2014.03.026, PMID: 24726698

[ref47] Magistrado-Coxen P. Aqeel Y. Lopez A. Haserick J. R. Urbanowicz B. R. Costello C. E.. (2019). The most abundant cyst wall proteins of *Acanthamoeba castellanii* are lectins that bind cellulose and localize to distinct structures in developing and mature cyst walls. PLoS Negl. Trop. Dis. 13:e0007352. doi: 10.1371/journal.pntd.0007352, PMID: 31095564 PMC6541295

[ref48] Mahboob T. Azlan A.-M. Tan T.-C. Samudi C. Sekaran S. D. Nissapatorn V.. (2016). Anti-encystment and amoebicidal activity of *Lonicera japonica* Thunb. and its major constituent chlorogenic acid in vitro. Asian Pac. J. Trop. Med. 9, 866–871. doi: 10.1016/j.apjtm.2016.07.008, PMID: 27633300

[ref49] Mazur T. Hadas E. Iwanicka I. (1995). The duration of the cyst stage and the viability and virulence of *Acanthamoeba* isolates. Trop. Med. Parasitol. 46, 106–108. PMID: 8525280

[ref50] Molet B. Ermolieff B. G. (1976). Description d'yne amibe d'eau douce: *Acanthamoeba* Lenticulata, Sp. Nov. (Amoebida). Protistologica 12, 571–576.

[ref51] Musgrave C. S. A. Fang F. (2019). Contact lens materials: a materials science perspective. Materials (Basel). 12:261. doi: 10.3390/ma12020261, PMID: 30646633 PMC6356913

[ref52] Neff R. J. (1957). Purification, axenic cultivation, and description of a soil amoeba, *Acanthamoeba* sp. J. Protozool. 4, 176–182. doi: 10.1111/j.1550-7408.1957.tb02505.x

[ref53] Oliveira G. Silva L. Leão T. Mougari S. da Fonseca F. G. Kroon E. G.. (2019). Tupanvirus-infected amoebas are induced to aggregate with uninfected cells promoting viral dissemination. Sci. Rep. 9:183. doi: 10.1038/s41598-018-36552-4, PMID: 30655573 PMC6336878

[ref54] Pharma Boardroom. Eyes on the prize: from Sicilian roots to FDA approval. Available at: https://pharmaboardroom.com/articles/eyes-on-the-prize-from-sicilian-roots-to-fda-approval/ (Accessed November 22).

[ref55] Primary Care Optometry News (2014). Experts agree on need to standardize testing for contact lens products. Available at: https://www.healio.com/news/optometry/20150420/j722_1911_1_news_print_1 (Accessed September 21, 2022).

[ref56] Pushkarew B. M. (1913). Über die Verbreitung der Süsswasser-Protozoen durch die Luft. Arch. Protistenkd. 23, 323–326.

[ref57] Randag A. C. van Rooij J. van Goor A. T. Verkerk S. Wisse R. P. L. Saelens I. E. Y.. (2019). The rising incidence of Acanthamoeba keratitis: a 7-year nationwide survey and clinical assessment of risk factors and functional outcomes. PLoS One 14:e0222092. doi: 10.1371/journal.pone.0222092, PMID: 31491000 PMC6731013

[ref58] Rayamajhee B. Willcox M. D. Henriquez F. L. Petsoglou C. Carnt N. (2021). Acanthamoeba keratitis: an increasingly common infectious disease of the cornea. Lancet Microbe. 2, e345–e346. doi: 10.1016/S2666-5247(21)00093-8, PMID: 35544193

[ref59] Robinson M. D. McCarthy D. J. Smyth G. K. (2009). edgeR: a Bioconductor package for differential expression analysis of digital gene expression data. Bioinformatics 26, 139–140. doi: 10.1093/bioinformatics/btp616, PMID: 19910308 PMC2796818

[ref60] Rolland S. Mengue L. Noël C. Crapart S. Mercier A. Aucher W.. (2020). Encystment induces Down-regulation of an acetyltransferase-like gene in *Acanthamoeba castellanii*. Pathogens 9:321. doi: 10.3390/pathogens9050321, PMID: 32357498 PMC7281194

[ref61] rrwick/Porechop, Github.Com (2017). Available at: https://github.com/rrwick/Porechop (Accessed September 26, 2022).

[ref62] Sawyer T. K. (1971). Acanthamoeba griffini, a new species of marine amoeba. J. Protozool. 18, 650–654. doi: 10.1111/j.1550-7408.1971.tb03391.x

[ref63] Sawyer T. K. Visvesvara G. S. Harke B. A. (1977). Pathogenic amoebas from brackish and ocean sediments, with a description of *Acanthamoeba hatchetti*, n. sp. Science 196, 1324–1325. doi: 10.1126/science.867031, PMID: 867031

[ref64] Schaap P. (2011). Evolutionary crossroads in developmental biology: *Dictyostelium discoideum*. Development 138, 387–396. doi: 10.1242/dev.048934, PMID: 21205784 PMC3014629

[ref65] Seal D. V. Bennett E. S. McFadyen A. K. Todd E. Tomlinson A. (1995). Differential adherence of *Acanthamoeba* to contact lenses: effects of material characteristics. Optometry Vision Sci. 72, 23–28. doi: 10.1097/00006324-199501000-00005, PMID: 7731652

[ref66] Siddiqui R. Khan N. A. (2012). Biology and pathogenesis of Acanthamoeba. Parasit. Vectors 5:6. doi: 10.1186/1756-3305-5-6, PMID: 22229971 PMC3284432

[ref67] Szentmary N. Daas L. Shi L. Laurik K. L. Lepper S. Milioti G.. (2019). Acanthamoeba keratitis - clinical signs, differential diagnosis and treatment. J. Curr. Ophthalmol. 31, 16–23. doi: 10.1016/j.joco.2018.09.008, PMID: 30899841 PMC6407156

[ref33] Shovlin, J. P. (2009). ”Testing efficacy against Acanthamoeba: the FDA should take into consideration the strength of the strain to be used as a standard, as well as how to make the testing scenario relevant” in Review of Optometry. Vol. 146. p. 113. Available at: https://galeapps.gale.com/apps/auth?userGroupName=tel_oweb&sid=googleScholar&da=true&origURL=https%3A%2F%2Fgo.gale.com%2Fps%2Fi.do%3Fid%3DGALE%257CA197442753%26sid%3DgoogleScholar%26v%3D2.1%26it%3Dr%26linkaccess%3Dfulltext%26issn%3D1930160X%26p%3DAONE%26sw%3Dw&prodId=AONE (Accessed November 30, 2022).

[ref68] Tu E. Y. Joslin C. E. (2010). Recent outbreaks of atypical contact lens-related keratitis: what have we learned? Am J. Ophthalmol. 150, 602–608.e2. doi: 10.1016/j.ajo.2010.06.045, PMID: 21036209 PMC2991148

[ref69] Vaillancourt B. Buell C. R. (2019). High molecular weight DNA isolation method from diverse plant species for use with Oxford Nanopore sequencing. bioRxiv [Preprint]. doi: 10.1101/783159

[ref70] Verani J. R. Lorick S. A. Yoder J. S. Beach M. J. Braden C. R. Roberts J. M.. (2009). National outbreak of Acanthamoeba keratitis associated with use of a contact lens solution, United States. Emerg. Infect. Dis. 15, 1236–1242. doi: 10.3201/eid1508.090225, PMID: 19751585 PMC2815976

[ref71] Walker B. J. Abeel T. Shea T. Priest M. Abouelliel A. Sakthikumar S.. (2014). Pilon: an integrated tool for comprehensive microbial variant detection and genome assembly improvement. PLoS One 9:e112963. doi: 10.1371/journal.pone.0112963, PMID: 25409509 PMC4237348

[ref72] Walters R. Campolo A. Miller E. McAnally C. Gabriel M. Shannon P.. (2022). Differential antimicrobial efficacy of preservative-free contact lens disinfection systems against common ocular pathogens. Microbiol Spectr. 10:e0213821. doi: 10.1128/spectrum.02138-21, PMID: 35138157 PMC8826922

[ref74] Wesley G. Giedd B. Hines B. Bickle K. Pearson C. Lorentz H. (2022). Safety and efficacy of a new water gradient biomimetic monthly replacement spherical contact lens material (Lehfilcon a). Clin. Ophthalmol. 16, 2873–2884. doi: 10.2147/OPTH.S362926, PMID: 36065354 PMC9440676

[ref75] Yoder J. S. Verani J. Heidman N. Hoppe-Bauer J. Alfonso E. C. Miller D.. (2012). *Acanthamoeba* keratitis: the persistence of cases following a multistate outbreak. Ophthal. Epidemiol. 19, 221–225. doi: 10.3109/09286586.2012.681336, PMID: 22775278

