# Supplementary material for: Acanthamoeba spp. aggregate and encyst on contact lens material increasing resistance to disinfection
Source: Front Microbiol. 2022 Dec 19;13:1089092. doi: 10.3389/fmicb.2022.1089092 (PMC9806144; doi:10.3389/fmicb.2022.1089092)
Supplement: Supplementary file 1 [file Data_Sheet_1.DOCX]

Supplementary Material

# Supplementary Data

Supplementary Material should be uploaded separately on submission. Please include any supplementary data, figures and/or tables. All supplementary files are deposited to FigShare for permanent storage and receive a DOI.

Supplementary material is not typeset so please ensure that all information is clearly presented, the appropriate caption is included in the file and not in the manuscript, and that the style conforms to the rest of the article. To avoid discrepancies between the published article and the supplementary material, please do not add the title, author list, affiliations or correspondence in the supplementary files.

# Supplementary Figures and Tables

## Supplementary Figures


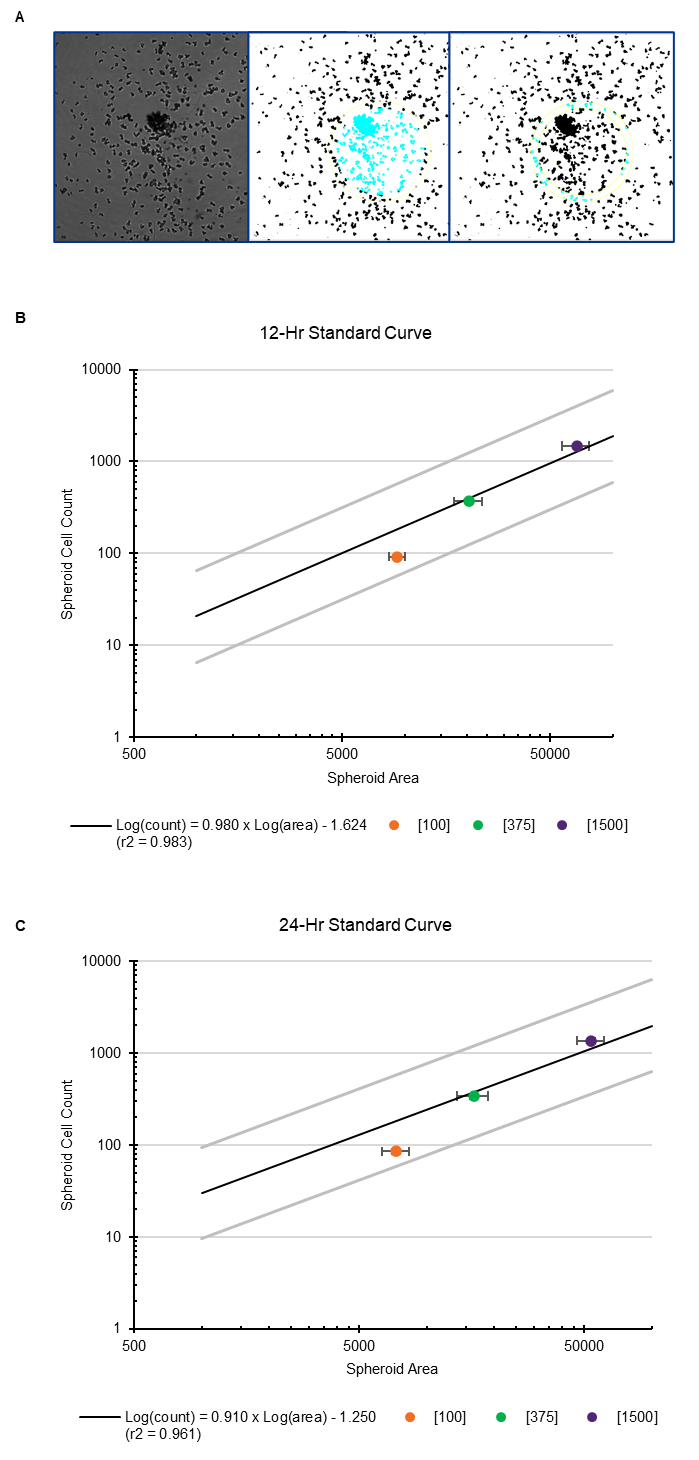


**Supplementary Figure 1.** Methodology for generating cell count estimates within spheroids. **A)** Representative image of left: beginning of spheroid formation, middle: area of cells which will be eventually included in spheroid as it accumulates, and right: counting cells which cross the perimeter threshold that encompasses the final spheroid. **B)** Hemocytometer counting method plotted against the 12 hour standard curve for cell count estimates. **C)** Hemocytometer counting method plotted against the 24 hour standard curve for cell count estimates. Hemocytometer results fall with in ± 0.5 log of the standard curve indicating agreement between methods.

**
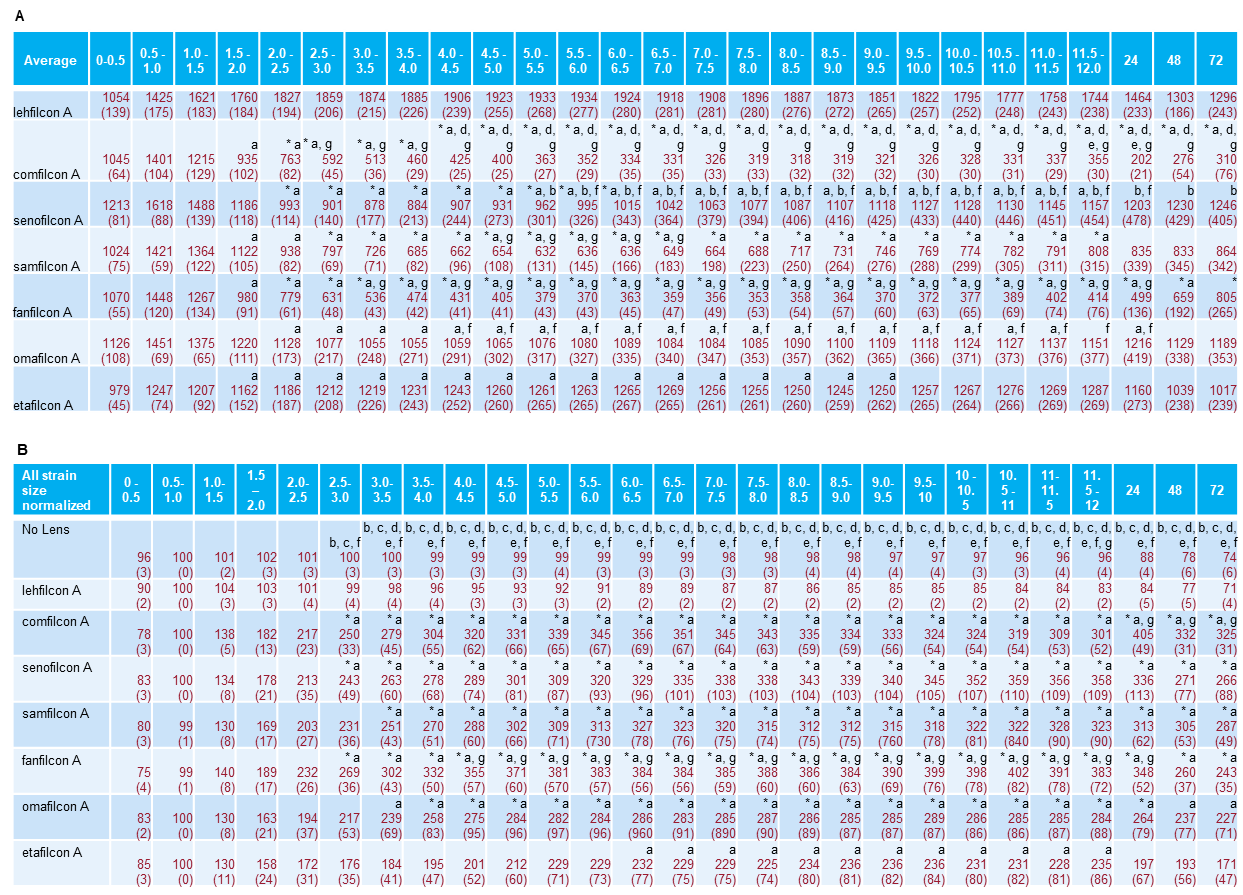
Supplementary Figure 2.** Statistical comparisons for Figure 2 between lenses of **(A)** all strains count (Figure 2A) and **(B)** all strains size (Figure 2B). Values of Figure 1 are noted as mean (SE). Size is normalized to the baselines obtained in the 0.5-1.0 hour. Analyzed via two-way repeat measure ANOVA. Within a given timepoint: *a* p < 0.05 vs lehfilcon A, *b* p < 0.05 vs comfilcon A, *c* p < 0.05 vs senofilcon A, *d* p < 0.05 vs omafilcon A, *e* p < 0.05 vs samfilcon A, *f* p < 0.05 vs fanfilcon A, *g* p < 0.05 vs etafilcon A. Within a given lens type, * p < 0.05 baseline (0.5-1.0 hour). Graphical representation noted in Figure 2. n=6 per group.

**
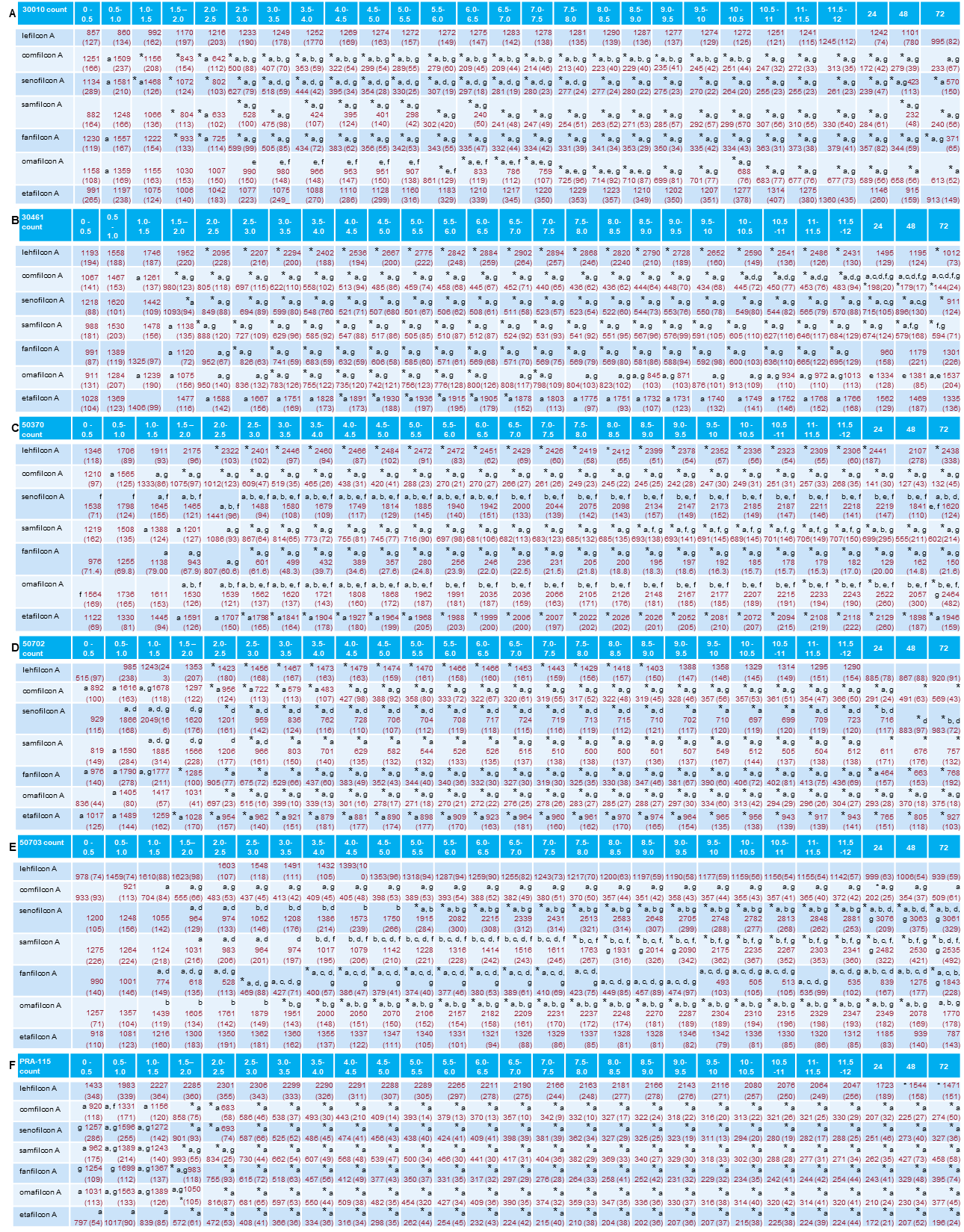
**

**Supplementary Figure 3.** Statistical comparisons for Figure 3 between lenses of of **(A)** ATCC 30010 count (Figure 3A), **(B)** ATCC 30461 count (Figure 3C), **(C)** ATCC 50370 count (Figure 3E), **(D)** ATCC 50702 count (Figure 3G), **(E)** ATCC 50703 count (Figure 3I), **(F)** ATCC PRA-115 count (Figure 3K). Values from Figure 3 are noted as mean (SE). Analyzed via two-way repeat measure ANOVA. Within a given timepoint: *a* p < 0.05 vs lehfilcon A, *b* p < 0.05 vs comfilcon A, *c* p < 0.05 vs senofilcon A, *d* p < 0.05 vs omafilcon A, *e* p < 0.05 vs samfilcon A, *f* p < 0.05 vs fanfilcon A, *g* p < 0.05 vs etafilcon A. Within a given lens type, * p < 0.05 baseline (0.5-1.0 hour). Graphical representation noted in Figure 3. n=6 per group.

**
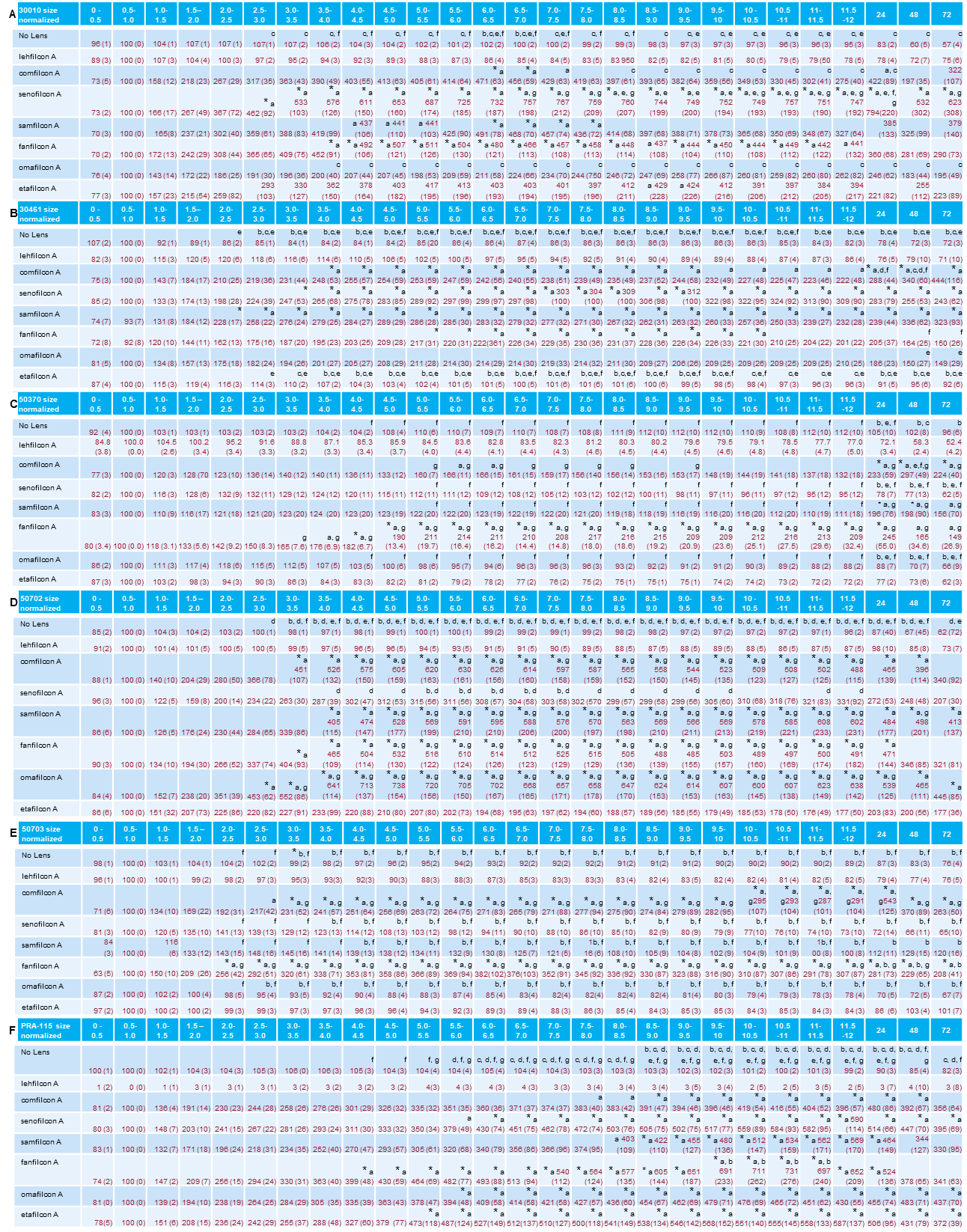
Supplementary Figure 4.** Statistical comparisons for Figure 3 between lenses of of **(A)** ATCC 30010 size (Figure 3B), **(B)** ATCC 30461 size (Figure 3D), **(F)** ATCC 50370 size (Figure 3F), **(H)** ATCC 50702 size (Figure 3H), (**J)** ATCC 50703 size (Figure 3J), **(L)** ATCC PRA-115 size (Figure 3L). Values from Figure 3 are noted as mean (SE). Size is normalized to the baselines obtained in the 0.5-1.0 hour. Analyzed via two-way repeat measure ANOVA. Within a given timepoint: *a* p < 0.05 vs lehfilcon A, *b* p < 0.05 vs comfilcon A, *c* p < 0.05 vs senofilcon A, *d* p < 0.05 vs omafilcon A, *e* p < 0.05 vs samfilcon A, *f* p < 0.05 vs fanfilcon A, *g* p < 0.05 vs etafilcon A. Within a given lens type, * p < 0.05 baseline (0.5-1.0 hour). Graphical representation noted in Figure 3. n=6 per group.

**Supplementary Video 1**. 72-hour timelapse video of *Acanthamoeba castellanii*, ATCC 30010 on lens materials.

**Supplementary Video 2**. 72-hour timelapse video of *Acanthamoeba polyphaga*, ATCC 30461 on lens materials.

**Supplementary Video 3**. 72-hour timelapse video of *Acanthamoeba castellanii,* ATCC 50370 on lens materials.

**Supplementary Video 4**. 72-hour timelapse video of *Acanthamoeba griffini* ATCC 50702 on lens materials.

**Supplementary Video 5**. 72-hour timelapse video of *Acanthamoeba lenticulate* ATCC 50703 on lens materials.

**Supplementary Video 6**. 72-hour timelapse video of *Acanthamoeba hatchetti* ATCC PRA-115 on lens materials.

**Supplementary Video 7**. Time-lapse video of an *Acanthamoeba* spheroid on lehfilcon A. Acanthamoeba spheroid created on comfilcon A, where it aggregated for 24 hours prior to moving to lehfilcon A. *Acanthamoeba* spheroid deaggregated over ~4 hours.
